# Temporal transcriptomic analysis using TrendCatcher identifies early and persistent neutrophil activation in severe COVID-19

**DOI:** 10.1172/jci.insight.157255

**Published:** 2022-04-08

**Authors:** Xinge Wang, Mark A. Sanborn, Yang Dai, Jalees Rehman

**Affiliations:** 1 Department of Biomedical Engineering, University of Illinois Colleges of Engineering and Medicine, Chicago, Illinois, USA.; 2 Department of Pharmacology and Regenerative Medicine and; 3 Division of Cardiology, Department of Medicine, University of Illinois College of Medicine, Chicago, Illinois, USA.

**Keywords:** COVID-19, Cell Biology, Immunology, Bioinformatics, Innate immunity

## Abstract

Studying temporal gene expression shifts during disease progression provides important insights into the biological mechanisms that distinguish adaptive and maladaptive responses. Existing tools for the analysis of time course transcriptomic data are not designed to optimally identify distinct temporal patterns when analyzing dynamic differentially expressed genes (DDEGs). Moreover, there are not enough methods to assess and visualize the temporal progression of biological pathways mapped from time course transcriptomic data sets. In this study, we developed an open-source R package TrendCatcher (https://github.com/jaleesr/TrendCatcher), which applies the smoothing spline ANOVA model and break point searching strategy, to identify and visualize distinct dynamic transcriptional gene signatures and biological processes from longitudinal data sets. We used TrendCatcher to perform a systematic temporal analysis of COVID-19 peripheral blood transcriptomes, including bulk and single-cell RNA-Seq time course data. TrendCatcher uncovered the early and persistent activation of neutrophils and coagulation pathways, as well as impaired type I IFN (IFN-I) signaling in circulating cells as a hallmark of patients who progressed to severe COVID-19, whereas no such patterns were identified in individuals receiving SARS-CoV-2 vaccinations or patients with mild COVID-19. These results underscore the importance of systematic temporal analysis to identify early biomarkers and possible pathogenic therapeutic targets.

## Graphical abstract

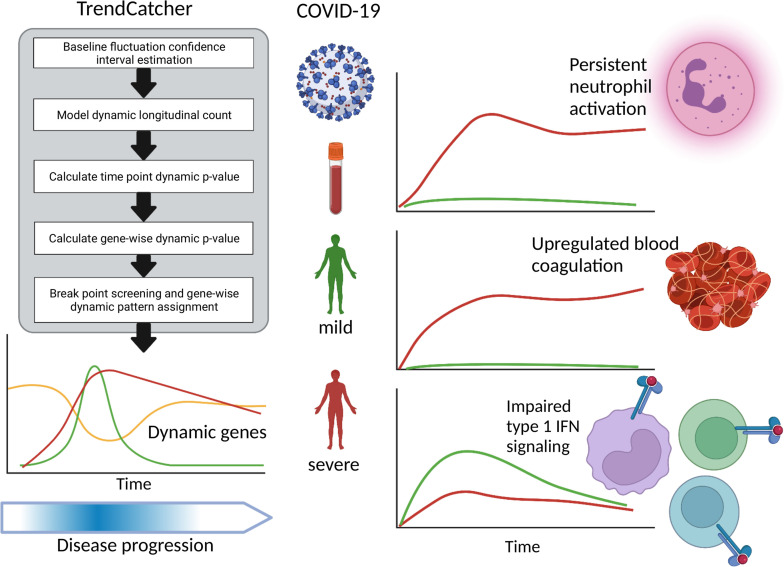

## Introduction

Time course transcriptomic profiling has been widely used to study and model dynamic biological processes in cells (1). By profiling mRNA levels during consecutive time points, researchers can infer dynamic responses to various external cues that cannot be observed by looking at only initial and terminal states. Recent improvements in high-throughput RNA-Seq technologies, including single-cell RNA-Seq (scRNA-Seq) provide viable approaches to study dynamic gene expression changes (2). scRNA-Seq especially allows for the in-depth analysis of temporal changes in distinct cell populations, thus providing insights into the heterogeneity and dynamics of responses to environmental cues or pathogenic stimuli. However, the analysis and visualization of longitudinal bulk RNA-Seq data or scRNA-Seq can be computationally challenging.

Currently, there are 2 predominant strategies for the analysis of sequential transcriptomic data sets. One strategy treats the sampling time points as categorical variables and is based on generalized linear models (GLMs). GLM-based packages include DESeq2 (3), edgeR (4), and limma (5). A complementary strategy is to treat time as a continuous variable and fit the time expression data into a spline-like model. These methods include DESeq2Spline (DESeq2 adopted with spline model for temporal RNA-Seq data sets) fitting, ImpulseDE2 (6) and Next maSigPro (7). The former strategies focus on the magnitude of change instead of the time order of gene expression, and they may also suffer from a relative loss of statistical testing power, especially if many time points are assessed (6). The latter strategy increases the power of detecting dynamic genes but is based on strong model assumptions that are not optimally suited for multiphasic responses, such as gene trajectory patterns that reflect initial acute stimuli, followed by counterregulatory compensatory responses. Furthermore, there is a lack of tools that leverage existing knowledge of functional pathway databases to infer and visualize pathway trajectories instead of individual gene trajectories only.

We found that this is challenging in a complex disease, such as COVID-19, caused by the SARS-CoV-2 infection (8). COVID-19 is characterized by distinct disease progression patterns that suggest diverse host immune responses (9). Patients with severe disease exhibit profound inflammatory responses and immunopathology (10). COVID-19 immunophenotyping studies involve a large number of time points from corresponding RNA-Seq and scRNA-Seq data sets (11, 12). Existing approaches for temporal transcriptomic analysis either do not take the temporal sequence into account when identifying dynamic differentially expressed genes (DDEGs) using pairwise comparison between time points or they do not systematically analyze pathways that are dysregulated at defined time points.

In this study, we developed TrendCatcher, an open-source R package (https://github.com/jaleesr/TrendCatcher) tailored for longitudinal bulk RNA-Seq and scRNA-Seq analysis. TrendCatcher uses a framework that combines the smooth spline ANOVA model and break point searching strategy, which identifies inflection points when gene expression trends reverse. We show that TrendCatcher outperformed commonly used methods for longitudinal RNA-Seq analysis when using simulated time course data for benchmarking. We also analyzed bulk RNA-Seq and scRNA-Seq gene expression profiles of peripheral blood cells in COVID-19 patients at various disease time points. TrendCatcher allowed us to identify and visualize dynamic gene expression signature profiles in peripheral blood that were associated with poor disease outcomes during the early phases of disease and could, thus, serve as potentially novel mechanistic targets, as well as early biomarkers for patient prognostication.

## Results

### TrendCatcher accurately identifies DDEGs in simulated data sets.

**Figure 1:**
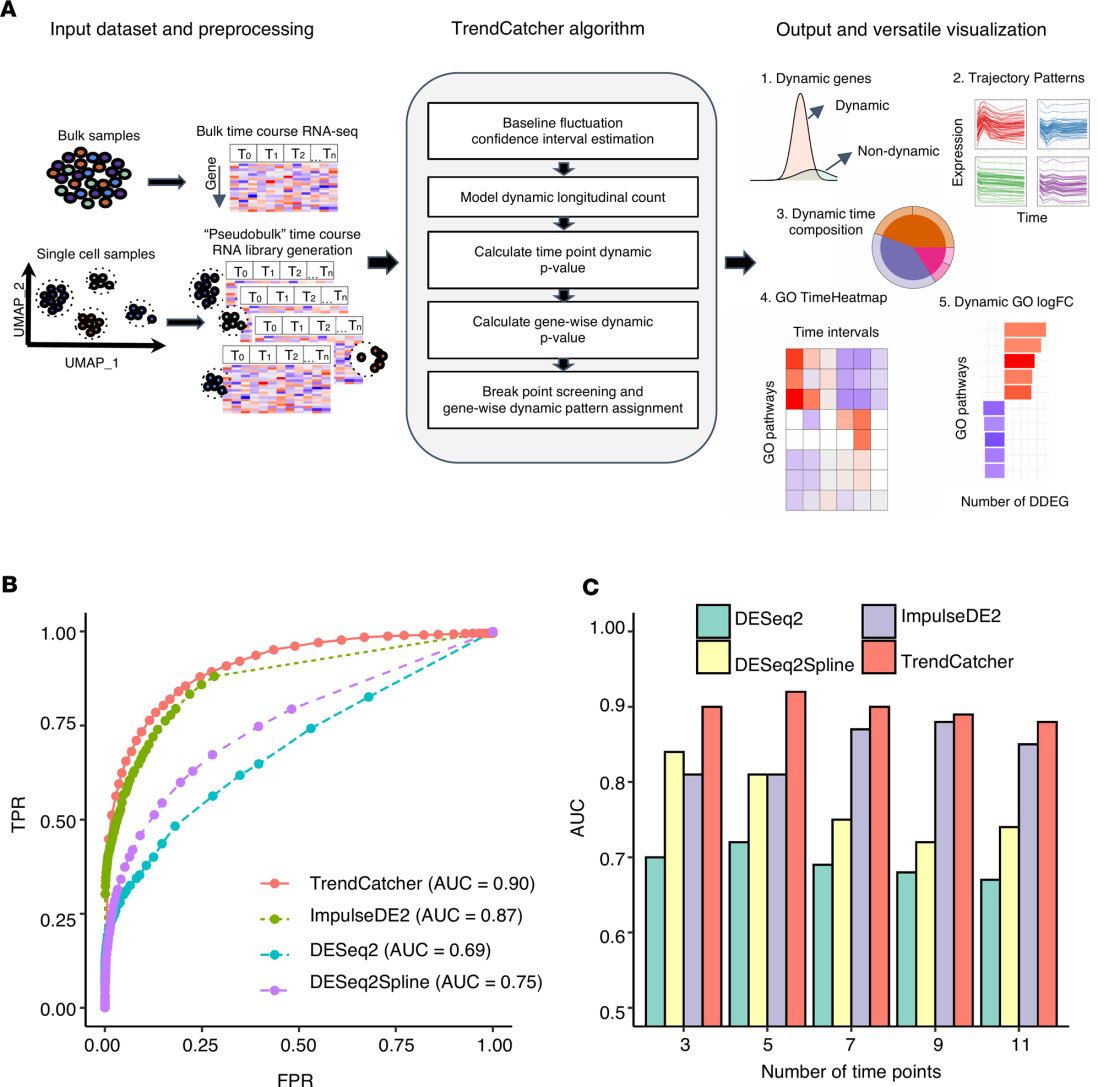
Overview and benchmarking of TrendCatcher. (**A**) TrendCatcher’s framework. TrendCatcher preprocesses input data, which includes creating cell type–specific “pseudobulk” data sets for temporal analysis when scRNA-Seq data is used. TrendCatcher’s core algorithm is composed of 5 main steps. TrendCatcher’s output includes 4 main types of visualizations and DDEGs identification (numbered 1–5). (**B**) TrendCatcher’s prediction ROC for a 7 time point–simulated data set compared with DESeq2, DESeq2Spline, and ImpulseDE2, with mixed trajectories. (**C**) TrendCatcher’s prediction performance (AUC) across different numbers of time points, from 3 to 11 time points. TrendCatcher’s AUC values across time points from 3 to 11 are 0.90, 0.92, 0.90, 0.89, and 0.88.

First, we tested the prediction performance of the TrendCatcher platform (Figure 1A) using a set of simulated time course RNA-Seq data sets because simulated data provide defined standards to assess the accuracy of analytical platforms. We considered a comprehensive collection of gene temporal trajectory patterns to simulate a set of realistic data with biological characteristics (1). We embedded 10,000 simulated trajectories with varied temporal patterns, including 90% nondynamic trajectories, 2.5% monotonous transition trajectories (continuously increasing or continuously decreasing gene expression levels throughout the time course), 2.5% impulse-shaped single–break point trajectories (only 1 temporal inflection point — i.e., up-peak-down or down-trough-up), 2.5% two–break point trajectories, and 2.5% three–break point trajectories (multimodal dynamic response — e.g., a combination of 2 or more basic types of trajectories). Compared with DESeq2, DESeq2Spline, and ImpulseDE2 using receiver operator characteristic (ROC), TrendCatcher had the largest AUC in a mixed simulated data set for time course data with 7 time points (Figure 1B). We also tested each model’s performance on a varying number of time points, including 3, 5, 7, 9, and 11 time points. As shown in Figure 1C, TrendCatcher had the highest prediction AUC across all time points, with the AUC values range from 0.88 to 0.90.

We next evaluated the prediction performance for each type of temporal trajectories. As shown in Supplemental Figure 1, A–C (supplemental material available online with this article; https://doi.org/10.1172/jci.insight.157255DS1), although the other 3 methods achieved slightly higher accuracy than TrendCatcher in monotonic trajectories, their AUCs dropped markedly when more complicated trajectories were embedded. DESeq2 only achieved AUC values of 0.49 to 0.62 for both biphasic trajectory and multimodal trajectory. The DESeq2Spline approach using a spline curve fitting model also dropped to an AUC of approximately 0.7 once multiphasic trajectories were introduced. These results suggest that existing approaches for longitudinal or time course analyses are well suited for monotonic trajectories (continuously up or continuously down) but that TrendCatcher may be more broadly applicable because it identifies monotonic, biphasic (up-down, down-up) and multiphasic shifts in gene expression, which are especially important in complex pathological setting when initial biological responses are followed by counterregulatory adaptive or maladaptive responses.

### TrendCatcher identifies rapid but transient upregulation of IFN signaling in peripheral blood following SARS-CoV-2 infection in a nonhuman primate model.

**Figure 2:**
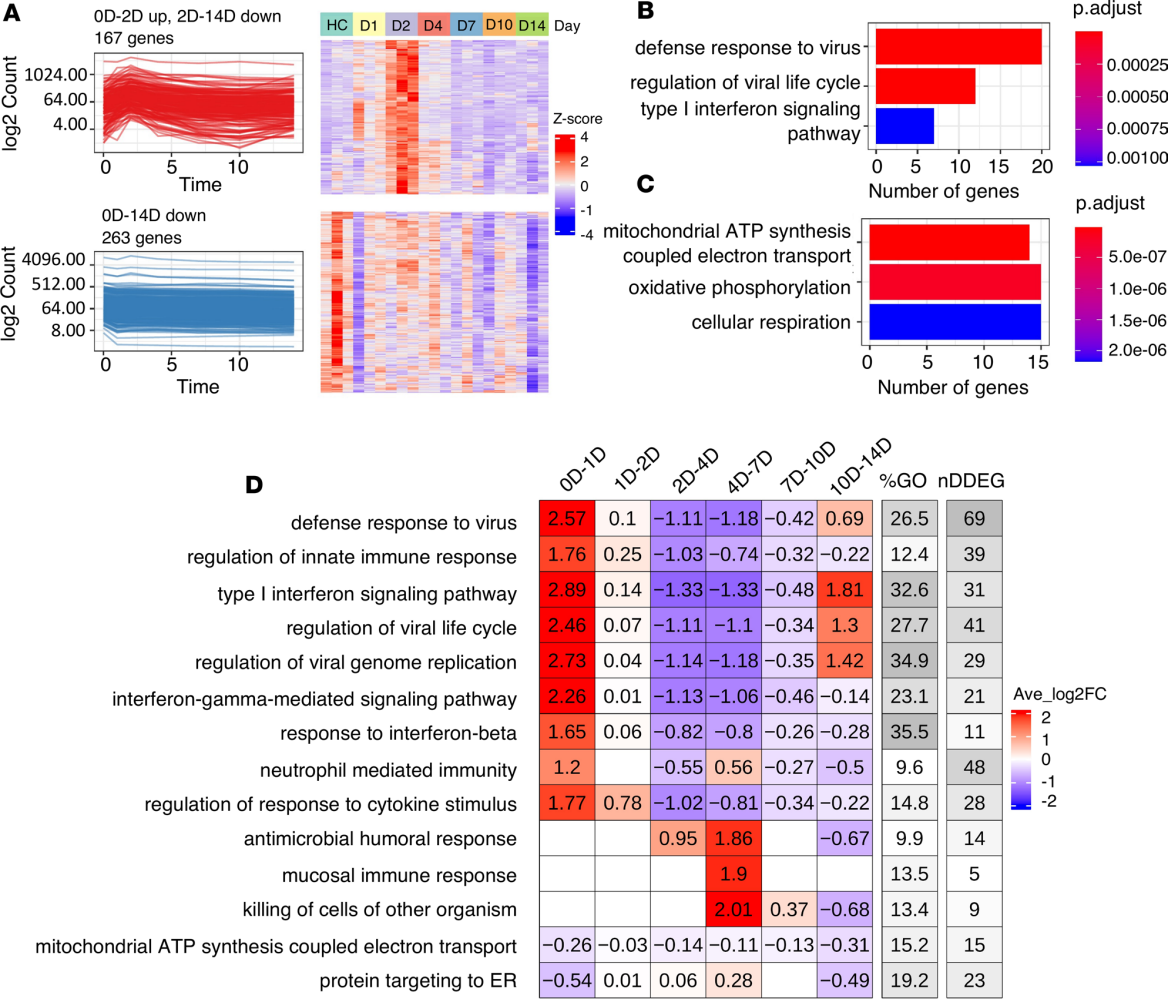
Dynamic gene expression in peripheral blood following SARS-CoV-2 inoculation in a nonhuman primate model. (**A**) Analysis of the 2 predominant trajectory patterns in the nonhuman primate peripheral blood RNA-Seq data from days 0 to 14. The top left figure represents 167 DDEGs following an up-down expression pattern, which peaked at day 2 and then slowly decreased until day 14. The top right figure represents their expression using a traditional *Z* score–normalized heatmap. The bottom left figure represents 263 DDEGs following a monotonic downregulated trajectory pattern, and their gene expression values were represented in the corresponding heatmap on the right. Gene expression values have been normalized and log_2_ transformed. (**B** and **C**) Top 3 GO enrichment analysis pathways using 167 DDEGs from trajectory pattern “0D-2D Up, 2D-14D Down” and 263 DDEGs from trajectory pattern “0D-14D Down”. The *x* axis represents the number of genes enriched in GO terms; the *y* axis represents the enriched GO terms; p.adjust represents adjusted *P* values using Holm-Bonferroni methods; and *P* values were generated by Fisher’s exact test. (**D**) TimeHeatmap of the top 15 dynamic pathways and their dynamic time windows visualizes the temporal patterns. Each column represents a time window. “0D-1D” represents days 0 and 1. The “%GO” column represents the percentage of DDEGs found in the corresponding pathway. The “nDDEG” column represents number of DDEGs found in the corresponding pathway. The number in each grid represents the Avg_log_2_FC of gene expressions compared with the previous time window. Color represents the Avg_log_2_FC of the DDEGs within each time window for the corresponding pathway.

To define the key dynamic gene signatures associated with SARS-CoV-2 infection in peripheral blood, we first analyzed the global transcriptomics profiles from a nonhuman primate data set (13), in which samples were collected on days 0 (uninfected controls), 1, 2, 4, 7, 10, and 14 following the live SARS-CoV-2 inoculation. This experiment in nonhuman primates had the advantage of clearly defining the timing of inoculation and following the time course of dynamic genes. TrendCatcher identified 962 DDEGs out of 12,754 total genes, accounting for 7.6% of total expression; this suggests that over 90% of the expressed genes in the peripheral blood remain close to the baseline expression levels even in the setting of the SARS-CoV-2 infection. We observed 2 major types of dynamic trajectories: (a) 167 genes followed a biphasic “0D-2D up, 2D-14D down” pattern, with their expression level peaking at day 2 and gradually returning close to baseline levels at day 14 (Figure 2A). These dynamic genes were primarily associated with host defense biological pathways annotated by gene ontology (GO), such as defense response to viruses, regulation of viral life cycle, and type I IFN (IFN-I) signaling pathways (Figure 2B). (b) Furthermore, 263 genes follow a “0D-14D down” pattern (Figure 2A). This set of genes followed a monotonous trajectory, with their expression gradually decreasing until day 14. Interestingly, we found these genes were primarily associated with mitochondrial ATP synthesis and oxidative phosphorylation, suggesting that disruption of mitochondrial respiration and ATP generation may be an important feature of the circulating cells’ transcriptomic shift in a SARS-CoV-2 infection (Figure 2C). TrendCatcher assigned trajectory pattern types to all dynamic genes and provided hierarchical pie charts to visualize the composition of trajectory patterns (Supplemental Figure 2A).

To systematically assess and visualize the dynamic programming of the top biological pathways associated with SARS-CoV-2 infection, TrendCatcher generated a TimeHeatmap. The TimeHeatmap function of TrendCatcher visualizes shifts in pathways by displaying the mean-fold change of individual DDEGs in a given pathway at defined time points, while also depicting the number of dynamic genes and the percentage of dynamic genes within that pathway (Figure 2D). This quantifies the pathway level shifts over time and allows for the magnitude of pathway change to be visualized, together with the number and fraction of dynamic genes in a given pathway, to gauge the relative importance of the pathway during the time course.

During initial infection (days 0–2), pathways related to innate immune response and IFN pathways were highly upregulated. Examples are upregulation of pathways such as defense response to virus and regulation of innate immune response increased with an average log_2_ fold change (log_2_FC) of 2.57 and 1.76 within the first day. Mucosal immune response, antimicrobial humoral response, and killing of cells of other organisms were activated during the later stage of infection (days 4–7), with an average log_2_FC around 2. On the other hand, mitochondrial ATP synthesis–coupled electron transport and protein targeting to ER, on the other hand, were gradually downregulated until day 14. Dynamic gene signatures from the IFN-I signaling pathway and mitochondrial ATP synthesis–coupled electron transport were shown using traditional heatmaps in Supplemental Figure 2, B and C. The temporal analysis of this nonhuman primate data set not only highlights the rapidity of IFN signaling activation, but also underscores that this initial burst of immune activation is transient and is followed by a gradual downregulation of the antiviral IFN responses during the first week following infection.

### Increased generation of immunoglobulin synthesis in plasma B cells as early as day 1 of symptom onset in patients diagnosed with SARS-CoV-2 infection.

**Figure 3:**
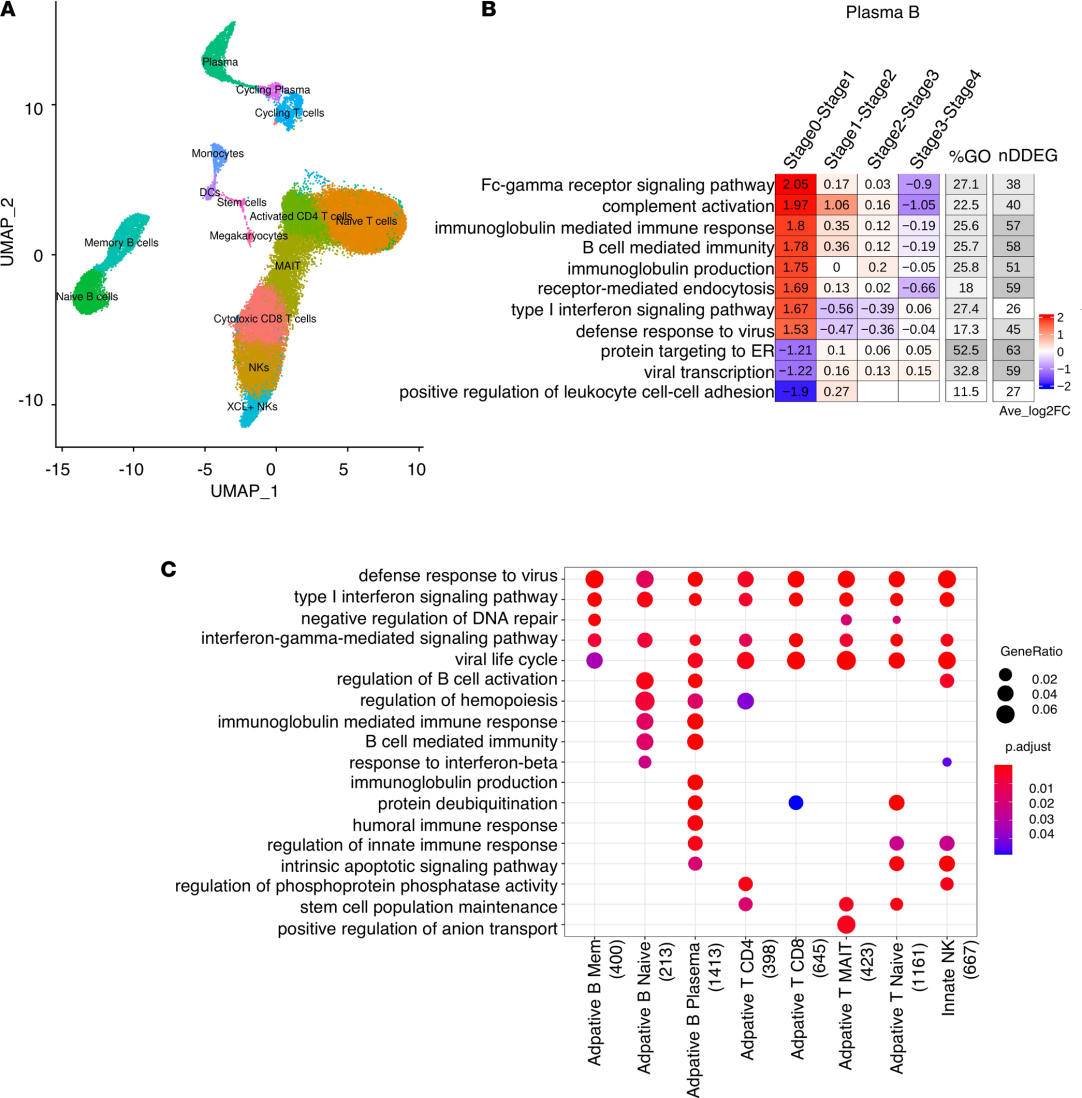
Cell type–specific dynamic gene expression in peripheral blood mononuclear cells following SARS-CoV-2 infection in patients. (**A**) UMAP visualization of scRNA-Seq PBMC data set (14) with annotated cell types from the original study. (**B**) TimeHeatmap of top dynamic biological pathway from plasma B cells. Each column represents a time window. Stage 0 represents uninfected baseline. The “%GO” column represents the percentage of DDEGs found in the corresponding pathway. The “nDDEG” column represents number of DDEGs found in the corresponding pathway. The number in each grid represents the Avg_log_2_FC of gene expressions compared with the previous time window. Color represents the Avg_log_2_FC of the DDEGs within each time window for the corresponding pathway. (**C**) Top GO enrichment comparison analysis using DDEGs from each cell type. The *x* axis represents cell types with the number of DDEGs shown in the brackets; the *y* axis represents the enriched GO terms; p.adjust represents adjusted *P* values using Holm-Bonferroni methods; and *P* values were generated by Fisher’s exact test. Dot size represents gene ratio.

Next, we analyzed the longitudinal gene expression profiles of peripheral blood mononuclear cells (PBMCs) obtained from patients diagnosed with a SARS-CoV-2 infection who were admitted to the hospital but predominantly had uncomplicated disease progression, with 4 of 5 patients showing only mild symptoms (14). The study performed scRNA-Seq analysis on PBMCs (Figure 3A), allowing for a cell type–specific analysis of gene expression shifts in distinct B cell subtypes, T cell subtypes, and NK cells. We adopted the cell type labels from the original study and generated pseudo-bulk RNA-Seq data sets for each cell type in order to quantify changes in DDEGs for a specific cell type. We also adopted the time annotation using stages, by binning the disease processes of COVID-19 patients from symptom onset to discharge into stages 0, 1, 2, 3, and 4. We defined stage 0 as the time point of samples obtained from healthy controls, and stages 1, 2, 3, and 4 represented days 1–16 from symptom onset. We only applied TrendCatcher to cell types containing more than 1000 cells at any given stage to ensure the robustness of the results.

TrendCatcher identified 400 DDEGs in memory B cells, 213 DDEGs in naive B cells, 1413 DDEGs in plasma B cells, 398 DDEGs in CD4^+^ T cells, 645 DDEGs in CD8^+^ T cells, 423 DDEGs in MAIT, 1161 DDEGs in naive T cells, and 667 DDEGs in NK cells. The TimeHeatmap of plasma B cells visualized the most dynamic biological pathways. Importantly, this temporal analysis of scRNA-Seq data shows how rapidly plasma B cells ramp up the upregulation of Fc-γ receptor signaling and immunoglobulin synthesis as early as stage 1 (which corresponds to day 1 of symptom onset) (Figure 3B). However, not all immunoglobulin genes are upregulated during the same temporal phase. As shown in Supplemental Figure 3, genes involved in immunoglobulin synthesis show distinct temporal patterns. Due to the comparatively lower number of plasma B cells, the prominence of such increased immunoglobulin changes may be diluted in peripheral blood bulk RNA-Seq analysis or PBMC bulk RNA-Seq analysis. However, temporal analysis of all PBMC types across the whole time course demonstrated increases in IFN-I signaling and defense responses to viruses as the most prominent changes over time (Figure 3C), thus mirroring the responses to SARS-CoV-2 we observed in the peripheral blood of nonhuman primates (Figure 2). To define cell type–specific temporal dynamics, we generated TimeHeatmaps for individual PBMC cell types and found significant upregulation of IFN-I signaling in T cells, NK cells, and memory B cells during stage 1 but subsequent downregulation by stage 2 of the disease in patients with mild symptoms (Supplemental Figure 4, A–C, and Supplemental Figure 5, A–D).

### TrendCatcher identifies early and persistent neutrophil activation as a hallmark of severe COVID-19.

**Figure 4:**
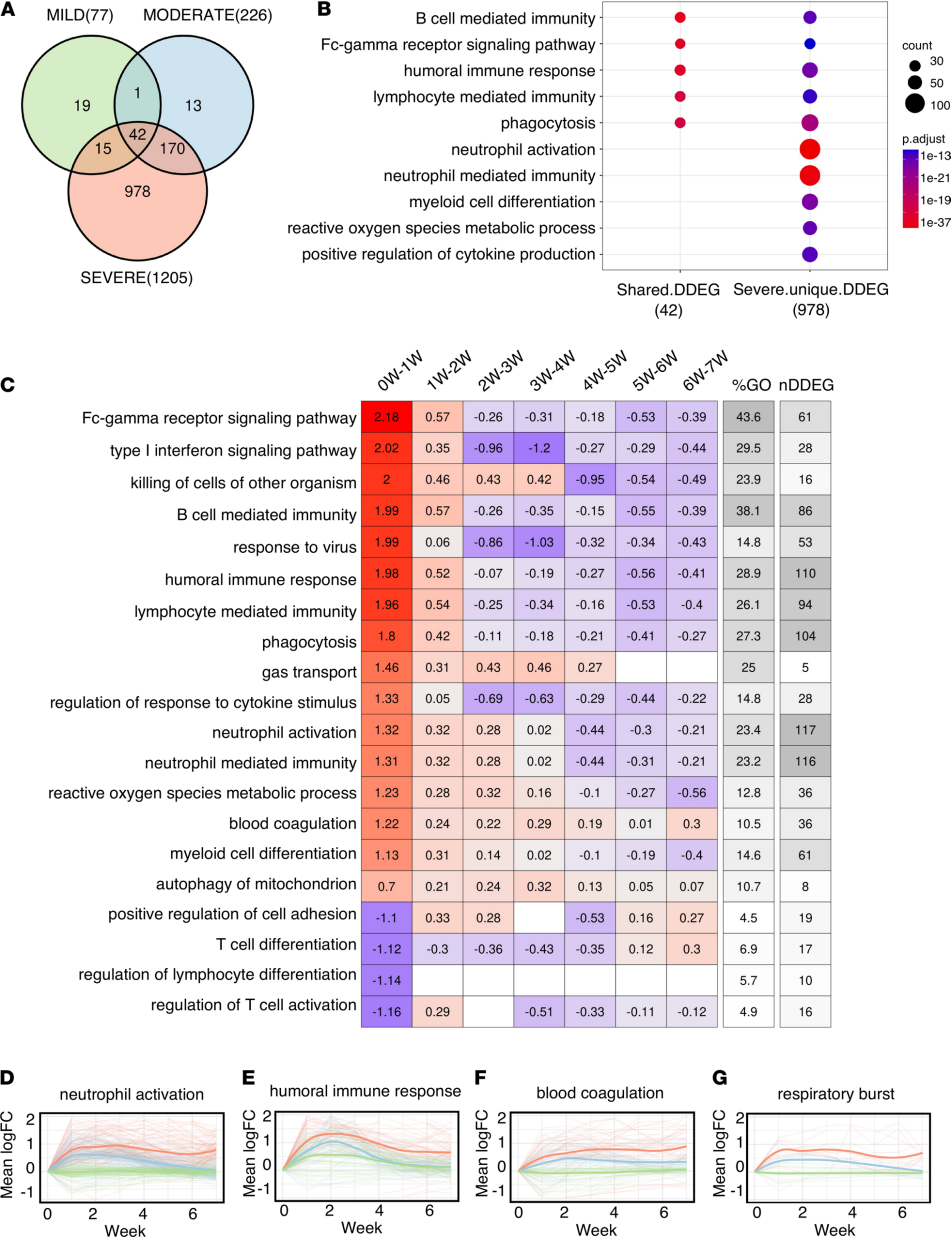
Temporal analysis of whole-blood RNA-Seq data in patients grouped according to disease severity. (**A**) Venn diagram of DDEGs identified from 3 COVID-19 severity groups, including mild, moderate, and severe. (**B**) Top GO enrichment from shared DDEGs across 3 groups compared with top GO enrichment from DDEGs only identified in severe group. The *x* axis represents comparison groups with the number of DDEGs shown in the brackets; the *y* axis represents the enriched GO terms; p.adjust represents adjusted *P* values using Holm-Bonferroni methods; and *P* values were generated by Fisher’s exact test. Dot size represents gene ratio. (**C**) TimeHeatmap of the top dynamic pathways from the severe group. Each column represents a time window. “0W-1W” represents week 0 (healthy control) to week 1. The “%GO” column represents the percentage of DDEGs found in the corresponding pathway. The “nDDEG” column represents number of DDEGs found in the corresponding pathway. The number in each grid represents the Avg_log_2_FC of gene expressions compared with the previous time window. Color represents the Avg_log_2_FC of the DDEGs within each time window for the corresponding pathway. (**D**–**G**) LOESS curve fitting of DDEGs identified in the severe COVID-19 group of the neutrophil activation pathway, humoral immune response pathway, blood coagulation pathway, and respiratory burst pathway. Red curves represent the severe group, blue curves represent the moderate group, and green curves represent the mild group. The *x* axis represents time in weeks; the *y* axis represents the Avg_log_2_FC of gene expressions compared with the baseline.

We then assessed whether a systematic temporal analysis of gene expression trends could be used to distinguish disease severity and prognosis of COVID-19 patients. We thus applied TrendCatcher to a time course human whole-blood bulk RNA-Seq data set (11), which contained longitudinal whole-blood transcriptomes from COVID-19 patients with mild, moderate, and severe clinical outcomes. TrendCatcher identified 77 DDEGs from the mild group, 226 DDEGs from the moderate group, and 1205 DDEGs from the severe group. Only 42 DDEGs were shared among these 3 groups (Figure 4A), and these were primarily associated with B cell–mediated immunity, Fc-γ receptor signaling pathways, humoral immune response, and lymphocyte mediated immunity (Figure 4B). However, TrendCatcher identified 978 DDEGs uniquely shown in the severe COVID-19 patient group. Importantly, these genes were strongly enriched for neutrophil-related biological pathways, including neutrophil activation and neutrophil-mediated immunity. These severe-disease–associated genes also included genes found in pathways such as myeloid cell differentiation, reactive oxygen species metabolic process, and positive regulation of cytokine production (Figure 4B). To compare how DDEGs and pathways identified by TrendCatcher compared with those identified by other platforms, we also applied 3 additional computational platforms (DESeq2, DESeq2Spline, and ImpulseDE2) to RNA-Seq data obtained from different COVID-19 severity groups and set the adjusted *P* value to 0.05 as a threshold to compare the DDEGs identified by different methods. As shown in the Venn diagram in Supplemental Figure 6, A–C, all methods showed that the number of identified DDEGs increased with disease severity and that the neutrophil activation pathway was highly enriched in the severe COVID-19 group when compared with the mild and moderate groups (Supplemental Figure 6, D–F). While there was broad consistency across platforms in the enriched pathways, the central advantage of the TrendCatcher approach is that it also identifies the time interval when the gene expression enrichment between groups begins to diverge.

To systematically characterize which biological processes in whole-blood RNA-Seq samples were most dynamic in severe COVID-19 patients and how they progress over time, we applied the TrendCatcher’s TimeHeatmap function. As seen in the nonhuman primate PBMC and the human COVID-19 scRNA-Seq data sets, we again observed the upregulation of genes involved in the response to virus, humoral immune response and IFN-I signaling pathway within week 1 of disease onset (Figure 4C). Importantly, some dynamic biological responses were only enriched in the group of patients that subsequently progressed to severe COVID-19, including neutrophil activation (117 DDEGs, 23.4% of the corresponding GO term), blood coagulation (36 DDEGs, 10.5% of the corresponding GO term), and regulation of response to cytokine stimulus and respiratory burst (Figure 4C and Supplemental Figure 7, A and B). For instance, neutrophil activation gene expression increased by a mean of 1.3 log_2_ units (approximately 2.5-fold increase in mean gene expression) within week 1, increased continuously until week 4, and only very gradually decreased in surviving patients by week 7. However, the summation of the averaged log_2_FC (Avg_log_2_FC) from the TimeHeatmap was larger than 0, which indicates that the neutrophil activation may not have returned fully to baseline levels by 7 weeks. We next applied LOESS smooth curve fitting to all the neutrophil activation DDEGs identified from the 3 severity groups, and we used a permutation test approach to quantify when and how the gene signatures differed significantly between groups. LOESS fitting confirmed that severe COVID-19 patients showed markedly high neutrophil activation at the early stage of infection (weeks 1 and 2), and also remained highly activated even after 7 weeks (Figure 4D). The permutation test function module in the TrendCatcher package identified the time interval when the differences between groups became apparent. We found that persistent neutrophil activation significantly separated the mild COVID-19 group from the severe COVID-19 group as early as the first time interval (days 0–7; Supplemental Figure 8A), whereas the difference in neutrophil activation between the moderate and severe group become apparent only at the beginning of week 2 (Supplemental Figure 8B).

Next, we combined TrendCatcher with the in silico cellular deconvolution tool MuSiC (15) to estimate cellular composition change over time, using a single-cell whole-blood data set (16) as a reference. As shown in Supplemental Figure 9, A and B, each stacked bar chart represents the cellular composition of a given sample. After quantifying estimated neutrophil percentages over time (Supplemental Figure 9C), we found an increase in the neutrophil percentage starting in week 1 in the severe COVID-19 group. We also applied TrendCatcher to the deconvoluted neutrophil gene expression profile using weighted cellular composition and observed persistent upregulation of neutrophil activation in the severe COVID-19 group (Supplemental Figure 9D), suggesting that the increase in neutrophil activation gene expression is driven by changes intrinsic to neutrophils.

From our bulk RNA analysis, we also observed that severe COVID-19 patients showed evidence of greater humoral immune response gene upregulation (Figure 4E), as well as markedly higher upregulation blood coagulation genes (Figure 4F) and respiratory burst genes (Figure 4G), which remained upregulated even after week 6. These were not found to be upregulated in patients with mild or moderate COVID-19 disease. These DDEGs and dynamic biological pathways may, thus, be suited to serve as early biomarkers that distinguish severe COVID-19 from mild and moderate COVID. All these dynamic gene signatures from neutrophil activation, blood coagulation, and respiratory burst pathways were listed using traditional heatmaps (Supplemental Figure 10 and Supplemental Figure 11, A and B).

### Early impaired IFN-I signaling in PBMC provides a hallmark of severe COVID-19.

**Figure 5:**
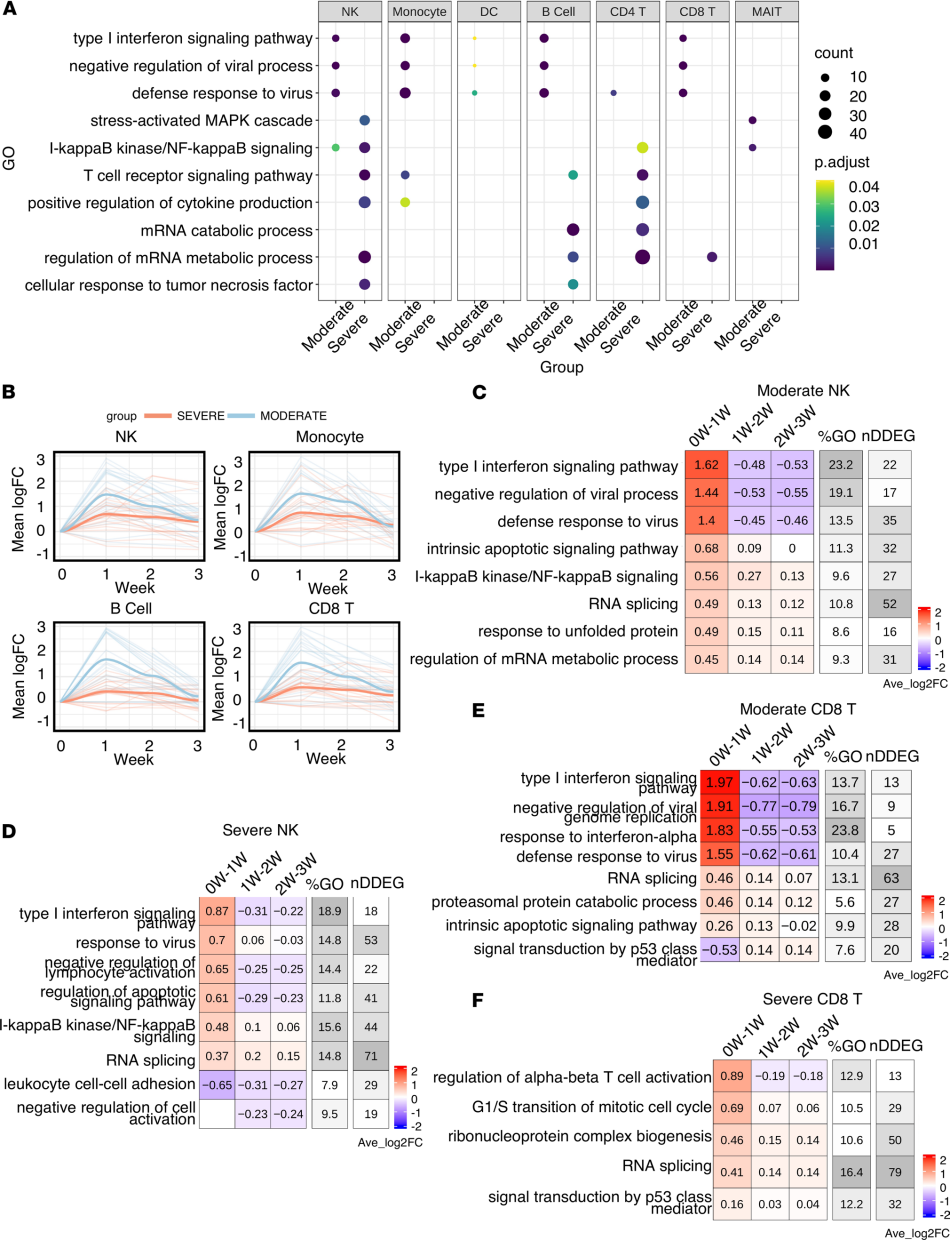
Temporal analysis of scRNA-Seq data of PBMCs from patients with either moderate or severe COVID-19. (**A**) Dot plot showing GO enrichment comparison between severe COVID-19 and moderate COVID-19 for each cell type. Each panel represents 1 cell type. The *x* axis represents severity group; the *y* axis represents the enriched GO terms; p.adjust represents adjusted *P* values using Holm-Bonferroni methods; and *P* values were generated by Fisher’s exact test. Dot size represents gene count. (**B**) LOESS curve fitting on DDEGs identified from the IFN-I pathway using TrendCatcher from moderate COVID-19 and severe COVID-19. Blue indicates moderate group, and red indicates severe group. The *x* axis represents time in weeks; the *y* axis represents the Avg_log_2_FC of gene expressions compared with the baseline. (**C**–**F**) TimeHeatmap of NK cells from moderate and severe COVID-19, CD8^+^T cells from moderate and severe COVID-19. Each column represents a time window. “0W-1W” represents week 0 (healthy control) to week 1. The “%GO” column represents the percentage of DDEGs found in the corresponding pathway. The “nDDEG” column represents number of DDEGs found in the corresponding pathway. The number in each grid represents the Avg_log_2_FC of gene expressions compared with the previous time window. Color represents the Avg_log_2_FC of the DDEGs within each time window for the corresponding pathway compared with the previous time window.

Next, to define the cell type–specific dynamic gene signatures and biological processes, we used TrendCatcher to analyze a human PBMC scRNA-Seq time course data set in which patients were categorized as having either moderate or severe COVID-19 (17). Importantly, since this data set only contained mRNA from PBMCs, it lacked mRNA from neutrophils. TrendCatcher generated “pseudo-bulk” mRNA profiles for each cell type in order to perform the analysis of gene expression dynamics in a cell type–specific manner. TrendCatcher identified more dynamic shifts in almost all cell types from severe COVID-19 patients compared with moderate groups (Supplemental Table 1 and Supplemental Table 2). To identify dynamic responsive processes unique to the severe group, we compared GO enrichment for each cell type between severe and moderate groups. As shown in Figure 5A, all innate immune cells and some adaptive immune cells (including B cells and CD8^+^ T cells) from moderate COVID-19 were highly enriched in IFN-I signaling, negative regulation of viral processes, and defense to viruses, whereas these pathways were not significantly enriched in severe COVID-19. Severe COVID-19 patients were highly dynamic in MAPK cascade, NF-κB signaling, T cell receptor signaling and positive regulation of cytokine production for both NK cells and CD4^+^ T cells. For monocytes and DC, no uniquely enriched dynamic biological processes were observed in severe COVID-19 patients versus moderate COVID-19 patients.

Furthermore, we also found the extent of the IFN signaling response to be the key distinguishing feature between moderate and severe COVID-19. To quantitatively compare the trajectory differentiation of the IFN-I signaling pathway, we performed the LOESS smooth fitting to the DDEGs identified from this pathway. As shown in Figure 5B, there is a profound separation between these 2 groups, with early strong activation of IFN-I signaling in NK cells, monocytes, B cells, and CD8^+^ T cells in moderate COVID-19 patients but not in severe COVID-19 patients. Patients with moderate COVID-19 showed activation of IFN-I signaling pathways, whereas patients with severe COVID-19 had a blunted activation of IFN-I signaling. This is also shown in the cell type–specific TimeHeatmap. As shown in Figure 5, C and D, although NK cells from both moderate and severe COVID-19 patients demonstrated activation of IFN-I response within the first week, moderate COVID-19 exhibited a stronger activation than the severe group, with average 1.62 log_2_FC compared with 0.87 log_2_FC. In CD8^+^ T cells, only moderate COVID-19 groups were observed to have a strong IFN-I response within the first week. On the other hand, CD8^+^ T cells in patients who would go on to develop severe COVID-19 showed upregulation of cell proliferation and cell differentiation genes, instead (Figure 5, E and F). These data suggest that a robust early IFN-I response in PBMCs is associated with reduced severity of COVID-19.

### TrendCatcher identifies metabolic gene expression shifts in NK cells as a hallmark response to COVID-19 vaccination.

**Figure 6:**
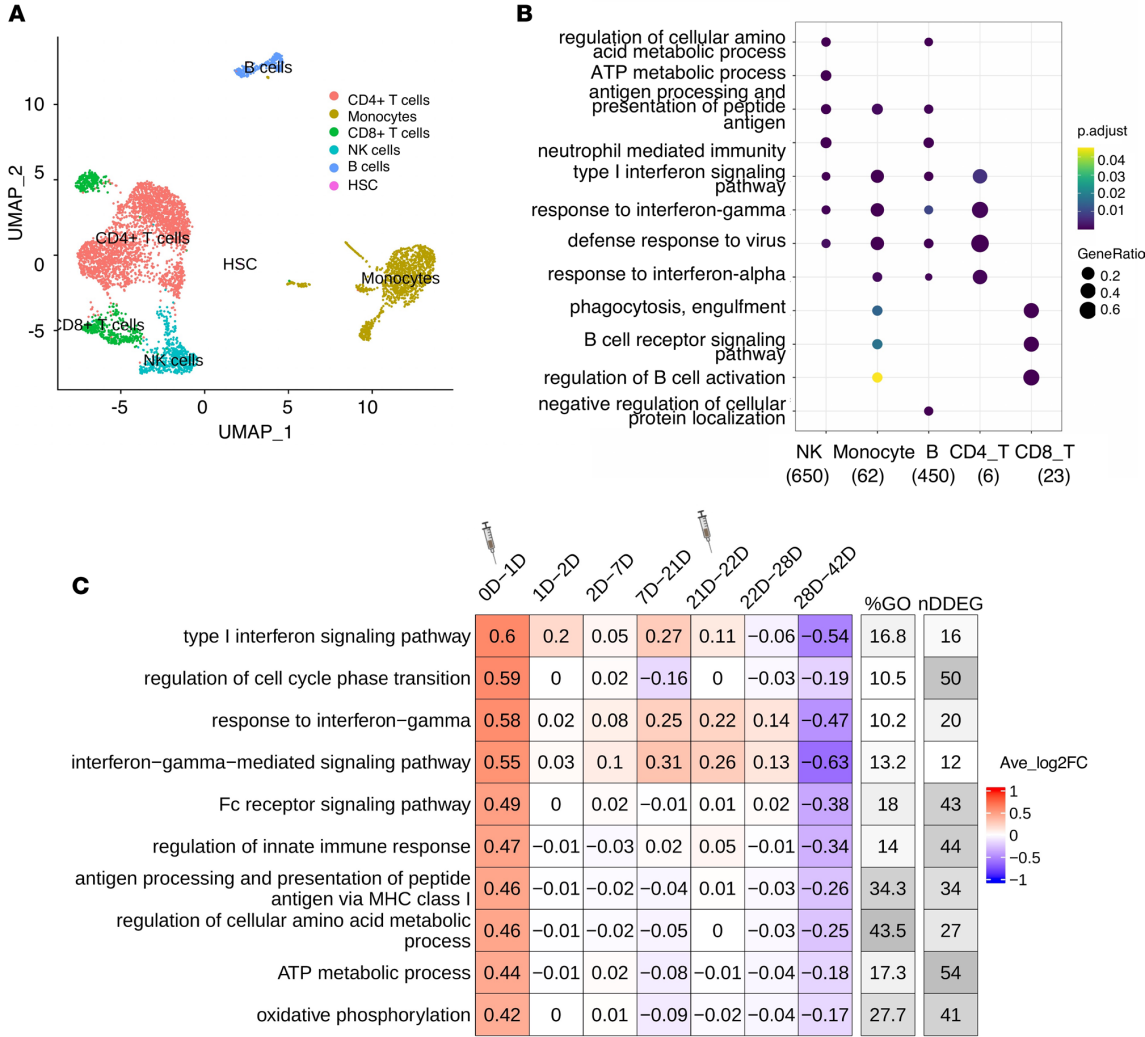
Temporal analysis of PBMC scRNA-Seq data from human subjects receiving the SARS-CoV-2 mRNA vaccine. (**A**) UMAP of the single-cell transcriptional profile of 1 patient on day 0. Cell types were autoannotated by SingleR. (**B**) Dot plot of comparison of the top GO terms enriched from cell type–specific DDEGs. The *x* axis represents cell type with the number of DDEGs shown in the brackets; the *y* axis represents the enriched GO terms; p.adjust represents adjusted *P* values using Holm-Bonferroni methods; and *P* values were generated by Fisher’s exact test. Dot size represents gene ratio. (**C**) TimeHeatmap of NK cells. Each column represents a time window. “0D-1D” represents day 0 (healthy control) to day 1. The “%GO” column represents the percentage of DDEGs found in the corresponding pathway. The “nDDEG” column represents number of DDEGs found in the corresponding pathway. The number in each grid represents the Avg_log_2_FC of gene expressions compared with the previous time window. Color represents the Avg_log_2_FC of the DDEGs within each time window for the corresponding pathway. The first dose was administered on day 1, and the second dose was administered on day 21.

We next applied TrendCatcher to a longitudinal human PBMC scRNA-Seq vaccination data set (18) to provide insight into how the immune system physiologically responds to mRNA vaccines over time. The vaccination study collected single-cell PBMCs from 56 healthy volunteers vaccinated with the Pfizer-BioNTech on days 0, 1, 2, 7, 21, 22, 28, and 42 after the vaccination. We processed each patient’s scRNA-Seq individually. We clustered cells using the Seurat algorithm (19) and annotated the cell types using SingleR (20). Then we remove cell types containing fewer than 1000 cells for each time point across all samples. As Figure 6A shows, it is the Uniform Manifold Approximation and Projection (UMAP) of 1 patient’s PBMCs single cell data on day 0.

TrendCatcher identified 650 DDEGs in NK cells, 450 in B cells, 23 in CD8^+^ T cells, 62 in monocytes, and only 6 in CD4^+^ T cells. This indicates that NK cells exhibit a strong dynamic gene expression shift after vaccination. After comparing the GO enrichment analysis across cell types, we found that NK cells gene expression shifts were enriched in metabolic processes in response to the Pfizer-BioNTech SARS-CoV-2 mRNA vaccine, such as regulation of cellular amino acid metabolism and ATP metabolism (Figure 6B). As shown in Figure 6C, a TimeHeatmap of NK cells shows 54 DDEGs, which account for nearly half of the ATP metabolic process pathway genes and exhibited a mean of 0.44 log_2_ fold upregulation after the first dose of vaccination. These findings indicate that the reprogramming of metabolism in NK cells may be an indicator of an intact vaccine response. Another hallmark of the vaccine response was the activation of IFN pathways, including IFN-I signaling pathway and response to IFN-γ (Supplemental Figure 12A). Importantly, the TimeHeatmap demonstrates that these gene expression shifts were very transient, usually decreasing by day 2 or day 7, and were again upregulated when the subjects received the booster vaccine at day 21. IFN pathways showed an average increase of 0.71 and 1.42 log_2_FC. In adaptive immune cells, we also observed strong IFN-I signaling pathways activated in both B cells and CD8^+^ T cells (Supplemental Figure 12, B and C). Additionally, B cells demonstrated upregulation of antigen processing and protein synthesis (Supplemental Figure 12B), which were upregulated only for the first day and then began decreasing. It is noteworthy that early upregulation of IFN pathways in the PBMCs of vaccine recipients mirrors that seen in patients with moderate COVID-19 infection, consistent with the notion that early and transient IFN upregulation is a hallmark of a healthy immune response to the SARS-CoV-2 infection.

## Discussion

Temporal analysis of gene expression is gaining importance in the analysis of complex dynamic processes such as disease progression. Besides gene dynamic pattern characterization, time course gene expression data are also used to infer regulatory and signaling relationships among genes (21, 22). Integrating with other different types of measurements, such as pathology and infection over time, helps disentangle the complex dynamic processes and possible underlying mediators (23). Thus, accurate identification of DDEGs in a time course RNA-Seq or scRNA-Seq study can help identify time-dependent disease mechanisms, adaptive and maladaptive molecular signatures, and potential biomarkers that may be associated with disease severity.

In recent years, tools have been implemented to characterize time course RNA-Seq data; however, these tools were focused on bulk RNA-Seq data sets (3, 6, 7), and few infer trajectories from scRNA-Seq (24, 25). Compared with methods that analyze time course data without considering the sequential nature of time points, modeling time as a continuous function avoids a relative loss of statistical testing power, especially when many multiple time points were studied. There is also a need for methods that combine DDEG identification with the visualization of dynamic pathway shifts.

In this study, we developed an open-source R software package to perform temporal analysis in longitudinal bulk RNA-Seq and scRNA-Seq data sets. TrendCatcher can identify DDEGs and infer the trajectories for each DDEG. By combining a time window sliding strategy on inferred gene trajectories and leveraging annotated biological pathway databases, TrendCatcher can infer and visualize the most dynamic biological pathways in response to the external stimuli. Using simulated data sets for benchmarking, TrendCatcher achieved higher accuracy (AUC = 0.9) compared with other commonly used methods for the analysis of temporal gene expression data sets, when analyzing 3 or more time points. When comparing our results with other methods using the whole-blood RNA-Seq data, we found that DESeq2 identified the fewest number of DDEGs in all patient groups, likely caused by a relative loss of statistical testing power by treating time as factorial variable. ImpulseDE2, identified the largest number of DDEGs, but around 50% of the DDEGs had only a 0.5 log_2_FC. This oversensitivity to gene expression changes of limited magnitude may be due to the strong model assumption, which is that the dynamic gene expression trajectory must either follow a monotonic curve or an impulse shape. However, none of the other 3 methods provided the output of gene expression estimation from replicates at each time point and also did not infer gene expression trajectories. As a result, these approaches cannot center a given gene’s expression level to its baseline, let alone compare their trajectories. In contrast, TrendCatcher provides an output of gene expression estimation from multiple replicates for each time point, which makes it possible for further trajectory comparison and further analysis, such as timing of the upregulation and downregulation of specific biological pathways. A key advantage of using TrendCatcher is identifying the time intervals when differences emerge. This is especially apparent in the setting of biphasic or multiphasic temporal trajectories, in which gene expression levels can change their trend of upregulation or downregulation during a time course. Importantly, by utilizing the TimeHeatmap function, TrendCatcher can help researchers identify the magnitude and dynamic nature of pathways shifts.

Despite the extraordinary success of rapidly developed and deployed mRNA vaccines against SARS-CoV-2, the ongoing COVID-19 pandemic remains a major global health problem, in part due to the emergence of newer highly contagious SARS-CoV-2 variants of concern, as well as vaccine hesitancy. This requires the identification of novel mechanistic targets, especially in vulnerable patients who have a high risk of developing severe COVID-19. One of the key pathogenic factors driving COVID-19 severity is the profound immune dysregulation observed in patients with severe COVID-19 that can result in respiratory failure (26–29). Human immune responses to SARS-CoV-2 infection are highly dynamic and time dependent, requiring upregulation, as well as downregulation, of distinct immune signaling pathways at the appropriate times to ensure optimal host defense (26–29). Understanding the dynamics of the COVID-19 immune response could form the basis of developing therapies that are appropriate for a given time window (30). Personalized medicine or precision medicine are gaining traction by developing therapies tailored to patients based on their genotype and phenotype, but personalization likely also requires tailoring therapies based on the temporal phase of a disease. These studies highlight the need for time course or longitudinal analyses of the host responses to the SARS-CoV-2 infection.

In this study, we applied TrendCatcher to systematically analyze sequential blood samples from either nonhuman primates infected with SARS-CoV-2, human patients with COVID-19 of varying severity, or human subjects who received a SARS-CoV-2 mRNA vaccine. TrendCatcher identified dynamic gene expression and biological pathway signatures for (a) SARS-CoV-2 infection progression over time, (b) severe COVID-19 versus moderate or mild COVID-19, and (c) COVID-19 vaccine recipients versus control. TrendCatcher established response to virus, humoral immune response, and IFN-I signaling pathway activation across peripheral blood cell types in mild, moderate, and severe COVID-19. However, we found temporal patterns of gene expression shifts that were unique in the severe COVID-19 patients. Severe COVID-19 was associated with marked activation of neutrophils and upregulation of coagulation pathways, as well as with blunted IFN-I signaling as early as week 1 in the peripheral blood of patients. Importantly, severe COVID-19 was associated with persistent activation of neutrophils and genes regulating coagulation for as long as 6 weeks, underscoring that the importance of the temporal analysis by TrendCatcher, which identified hallmarks of severe COVID-19 in peripheral blood samples. It is important to note that, while peripheral blood samples are convenient to obtain for longitudinal studies because they only involve minimally invasive blood draws, they give a limited picture of the gene expression and activation states because gene expression patterns of immune cells in a tissue may be different from their counterparts in the circulating blood.

Our findings complement recent studies that implicate neutrophils in the excessive inflammation, coagulopathy, immunothrombosis, and organ damage that characterize severe COVID-19 (31–35). Neutrophils are particularly active in highly vascularized organs, such as lungs and kidneys, which are prime targets of SARS-CoV-2–induced injury in COVID-19 (36). Dysregulated neutrophil responses, such as prolonged activation, may cause damage to vessels and underlying parenchymal (37–39). Studies also show that activated neutrophils through TLRs, chemokine receptors, and cytokine receptors can stimulate the neutrophil extracellular trap (NET) formation (31). Furthermore, recent studies showed that the disbalance between NET formation and degradation can trigger immunothrombosis and tissue injury (40, 41). Excessive activation of macrophages and adaptive immune cells in severe COVID-19, which can form vicious cycles of positive feedback circuits, has also been demonstrated (42, 43), but less is known about how early neutrophils are activated in disease progression because many of the studies on neutrophils in clinical COVID-19 or animal models of severe SARS-CoV-2 focused on the late stages of the disease, as well as the neutrophils in the lung tissue. In our study, we used a systematic temporal analysis and observed that profound neutrophil activation in the peripheral blood, which was predominant in the severe COVID-19 group, occurs as early as week 1 after diagnosis, and we observed that symptoms persist even after 6 or 7 weeks in surviving patients.

Our observation of early upregulation of coagulation genes in the whole-blood transcriptomes of severe COVID-19 patients also points to another feature that is associated with severe COVID-19, thrombotic, and embolic complications such as strokes (13, 44, 45). Recent studies have found that the procoagulant changes in endothelial cells underly the coagulopathy in severe COVID-19 (46). Endothelial dysfunction in COVID-19 patients may be exacerbated through inflammatory cytokines and NETs, thus pointing to interactions between circulating or recently recruited neutrophils and the vessel wall endothelial cells that are in contact with circulating immune cells as drivers of such coagulopathic manifestations (47–49). We believe the analysis of the RNA-Seq and scRNA-Seq data support neutrophil activation and upregulation of coagulation genes as defining features of severe COVID-19, highlighting their role as early biomarkers to provide prognostic information and, thus, optimally treat patients who have a higher likelihood of progressing to severe disease. Importantly, our results also raise questions about the role of neutrophils and of coagulation in what is referred to as “long COVID” or “postacute sequelae of COVID” (50–52) because we observed that the activation persists for as long as 6–7 weeks after the initial infection.

Our temporal analysis showed that severe COVID-19 single-cell PBMCs were characterized by impaired IFN-I signaling at the onset of infection (week 1), compared with the moderate COVID-19 group. This impaired IFN-I signaling was identified in innate cells, such as NK cells and monocytes, and adaptive cells, such as B cell and CD8^+^ T cells. IFN-I, which are essential for antiviral immunity (53, 54), have been shown to be upregulated in COVID-19 (55, 56). Other studies also suggested that impaired IFN-I signaling may promote severe COVID-19 and that IFN therapy could be used as therapy in severe COVID-19 (57). However, since these studies were not designed for longitudinal analyses, the timing of when to intervene on IFN signaling was not clear. Traditional analyses of gene expression data are not suited for the identification of temporal windows and, thus, do not address whether impaired IFN production is present at the onset of infection, whether it was delayed, or whether IFN production is exhausted after an initial activation (56). TrendCatcher addressed this question by showing the dynamic timing of type I IFN from both the moderate group and severe group.

In our COVID-19 vaccination single-cell PBMC temporal data analysis, we also identified prominent metabolic shifts in NK cells. Cellular metabolism is recognized as an important factor that can determine the fate and function of lymphocytes (58, 59). Certain metabolic pathways have been shown to have direct immunoregulatory roles (58). Activated NK cells engage in a robust metabolic response, which is required for normal effector functions (60), and our data suggest that assessing NK metabolic shifts may be an intriguing alternative to assessing vaccine responsiveness; this does not rely on antibody levels, which can be highly variable in laboratory assays.

In conclusion, we have developed the potentially novel TrendCatcher R package platform designed for time course RNA-Seq data analysis, which identifies and visualizes distinct dynamic transcriptional programs. When applied to real whole-blood bulk RNA-Seq time course data sets from COVID-19 patients, we observed that patients with severe COVID-19 showed gene expression profiles consistent with profound neutrophil activation and coagulopathy early during the progression of the disease (starting from the first week of symptom onset). Even though the application of TrendCatcher in this manuscript focused on COVID-19 data sets, it has been designed to be used for the analysis of a broad range of dynamic biological processes and diseases.

## Methods

### TrendCatcher framework.

The main components of the TrendCatcher framework are shown in Figure 1. TrendCatcher requires 2 main inputs: the raw count table C of a temporal study with a dimension of *m* × *n*, where *m* denotes the number of genes and *n* denotes the number of samples, and a user-defined baseline time variable T, such as “0 hour”. Since samples may have different sequencing depths and batch effect, TrendCatcher integrates with limma (5) and provides preprocessing steps, such as batch correction and normalization. For scRNA-Seq data sets, TrendCatcher extracts cells for each cell type annotated in the meta data slot of Seurat object and converts it into a cell type–specific “pseudobulk” time course RNA library. Based on a user-specified threshold, genes of relatively low abundance are removed from the count table, reads are normalized, and batch effects are removed. TrendCatcher’s core algorithm is composed of 5 main steps: (a) baseline fluctuation confidence interval estimation, (b) model dynamic longitudinal count, (c) time point dynamic *P* value calculation, (d) gene-wise dynamic *P* value calculation, and (e) break point screening and gene-wise dynamic pattern assignment. Mathematical details will be expanded in the following sections. For the output of TrendCatcher, there are mainly 2 components: a master table and a set of functions for versatile visualization purposes. The master table contains all the dynamic details of each single gene, including its dynamic *P* value, its break point location time, and its dynamic trajectory pattern. In addition to the master table, TrendCatcher produces 5 main types of visualizations: (a) a figure showing the observed counts and fitted splines of each gene, (b) genes trajectories from each trajectory pattern group, (c) a hierarchical pie chart that represents trajectory pattern composition, (d) a TimeHeatmap to infer trajectory dynamics of top dynamic biological pathways, and (e) a 2-sided bar plot to show the top most positively and negatively changed (averaged accumulative log_2_FC) biological pathways.

### Baseline fluctuation confidence interval estimation.

We assumed that the observed number of RNA-Seq reads count from the baseline time (e.g., t = 0 hour) *X_i,t_* *_baseline_* was generated from a negative binomial (NB) distribution. 

*X_i,t__baseline_~ NB*(*μ_i,t__baseline_,φ_i_*) (Equation 1)

Where *μ_i,t baseline_* is the mean count of gene *i* (*i* refers to the index of the gene) at the baseline time point, and *φ_i_* is the dispersion factor. First, the dispersion factor φ*_i_* was preestimated as a constant hyperparameter for each gene with DESeq2 (3), as shown in Equation 2. Here, *σ_i_(t)* is the variance at time t. 

*σ_i_*(*t*)*^2^* = μ_i_(*t*) + φ_i_ × μ(*t*)^2^ (Equation 2)

Then, *μ_i,t_* *_baseline_* was estimated using maximum likelihood from Equation 3. 


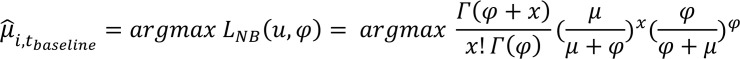
 (Equation 3)

Based on the NB distribution and the estimated mean count for baseline time, we constructed the 90% CI (*X_i,t baseline,0.05_*, *X_i,t baseline,0.95_*) as the baseline count fluctuation interval.


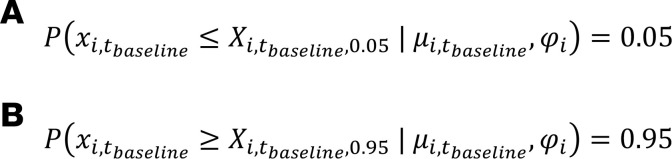
 (Equation 4A, 4B)

### Model dynamic longitudinal count estimation.

To model the time-dependent gene expression value, we applied a smoothing spline ANOVA model (61, 62) with a NB family constraint to fit the reads from samples across nonbaseline multiple time points. The random variable *X_i,t_* *_(t≠t_* *_baseline)_* is assumed to follow the NB distribution in Equation 5, with a positive integer α represents number of failures before the *α*th success in a sequential of Bernoulli trials, and *p*(*t*) ∈ (0,1) represents the success probability. 

*X_i,t__(t≠t__baseline)_* ~ *NB*(*α*,*p*[*t*]) (Equation 5)

The log-likelihood given a time course observed count x = {*x_i,t_* *_(t≠t_* *_baseline)_*}*_i =_*_1,...,n;_ *_t_* *_=_* *_1,... T_* is calculated as Equation 6. 


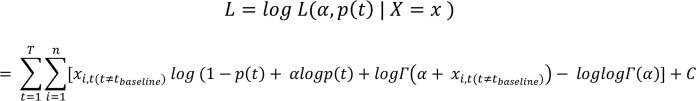
 (Equation 6)

Taking the logit link and model time effect, we define the logit link η.


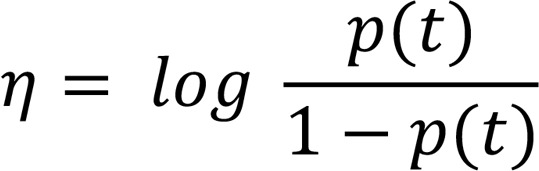
 (Equation 7)

To allow for flexibility in the estimation of η, and find the best trade-off between goodness of fit and the smoothness of the spline curve, soft constraints of the form *J*(*η*) is added to the minus log-likelihood, with the smoothing parameter λ > 0.

–*L* + *λ* J(η) (Equation 8)

The solution to the optimization of Equation 8 leads to a smoothing fitting to the reads from samples across different nonbaseline time points. The estimated mean of *μ*_i,t(t≠tbaseline)_ can be estimated using Equation 9.


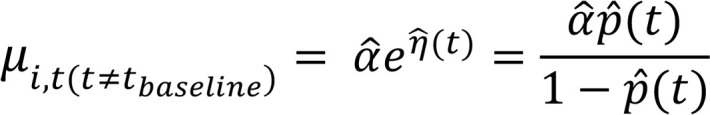
 (Equation 9)

### Gene’s dynamic P value calculation.

To calculate gene’s nonbaseline dynamic signal significance, each gene’s nonbaseline estimated mean count *μ*_i,t(t≠tbaseline)_ was tested against the baseline fluctuation interval. Based on Equation 10A and Equation 10B, for each gene at each single nonbaseline time point, a dynamic time *P* value was calculated.

*Ifμ*_i,t(*t*≠*tbaseline*)_ ≥ μ*_i,tbaseline_, then p_i,t_* = ∫*P*(*x*)*≥* μ_i,t(t≠tbaseline)_*|*μ*_i,tbaseline_,φ_i_*) (Equation 10A)

*Ifμ*_i,t(*t*≠*tbaseline*)_ ≤ μ*_i,tbaseline_, then p_i,t_* = ∫*P*(*x*)≤ μ_i,t(t≠tbaseline)_*|*μ*_i,tbaseline_,φ_i_*) (Equation 10B)

Then, we applied Fisher’s combined probability test method to calculate a gene-wise dynamic *P* value. 


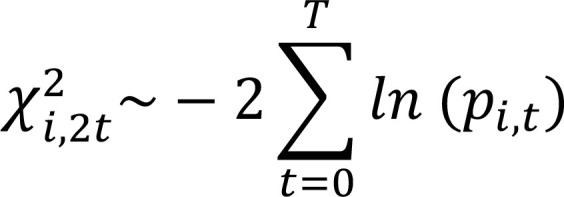
 (Equation 11)

### Trajectory pattern assignment.

First, we connect all the significant dynamic signal time points, with a *P* value threshold less than 0.05. Then, we applied a break point searching strategy to capture the gene expression change trend. The definition of break point is defined using Equation 12A and Equation 12B.

*Ifμ_i,t,_ >μ_i,tnext_ ANDμ_i,t_ >μ_i,tprevious_, break point type I* (Equation 12A)

*Ifμ_i,t,_ <μ_i,tnext_ ANDμ_i,t_ <μ_i,tprevious_, break point type II* (Equation 12B)

There are 2 types of break points: type I means a gene’s expression level is upregulated followed by a downregulation, and type II means a gene’s expression level is downregulated, followed by an upregulation. By screening along the break point, the master-pattern and subpattern were assigned to each gene.

### TimeHeatmap enrichment analysis and 2-sided bar plot.

To build the TimeHeatmap for visualizing the biological pathway enrichment change over time, we designed a window-sliding strategy to capture all the upregulated or downregulated genes within each time interval. If we denote time vector as *t_j_* and ∈ 1,…, *T*, each time interval is denoted as [*t_j-1_*, *t_j_*]. We found *N_up_* upregulated genes and *N_down_* downregulated genes within the time window [*t_j-1_*, *t_j_*]; then, Fisher’s exact test was performed to obtain the GO term enrichment with the corresponding time interval for upregulated genes and downregulated genes separately. Users can select the top most enriched biological pathways (ranked by enrichment *P* value) for each time interval (the default is top 10 most enriched pathways). Then, for each selected GO term within the corresponding time window, we calculated the averAvg_log_2_FC of all the DDEGs from this GO term. A series of Avg_log_2_FC values over time characterize the trajectory dynamics of the corresponding biological pathway; it is defined as biological pathway trajectory inference in this study. The summation of the series of Avg_log_2_FC estimates the averaged accumulative log_2_FC (GO_mean_logFC) for the corresponding GO term. TrendCatcher ranks biological pathways based on their dynamic magnitude inferred from the GO_mean_logFC value. Users are free to choose the top most positively and negatively changed (averaged accumulative log_2_FC) biological pathways. Besides GO enrichment analysis (63), TrendCatcher also packages Enrichr (64) biological pathway databases. Visualization is constructed using the ComplexHeatmap (65) package.

### Simulated data set.

To mimic the real biological RNA-Seq data set, we only allowed approximately 10% of the genes to be dynamic responsive genes. In this study, we embedded 5 different types of trajectories into the temporal RNA-Seq simulated data sets, including nondynamic trajectory (~90%), monotonic trajectory (~2.5%), biphase trajectory (~2.5%), and multimodal trajectory (~5%), including 2–break point and 3–break point trajectory. Each type of trajectory was constructed by adding NB distribution noise to the embedded trajectory count. For example, for monotonic trajectory, we defined the first and last time points’ RNA expression level changes to be 0.5–2 log_2_FC. Then, we added NB distribution noise to the embedded monotonic trajectory. To check how the AUC changes as time course study extends longer, we sampled 3, 5, 7, 9, and 11 different numbers of time points with even time intervals, and we randomly sampled 3 replicates for each time point. To validate how the prediction AUC varies across different types of trajectories, we embedded approximately 10% of the genes to have nondynamic trajectories, and approximately 90% of the genes have a specific dynamic trajectory (monotonic, biphase, or multimodal).

### Pseudobulk RNA library construction.

To construct “pseudo-bulk” RNA library from scRNA-Seq data sets, all cells for each cell type in a given sample were computationally “pooled” by summing all counts for a given gene. Since pseudobulk libraries composed of few cells are not likely modeled properly, we removed cell types containing less than 1000 cells in this study. Lowly expressed genes were removed for each cell type, as well, using the filterByExpr function from edgeR R package (4). Gene counts were transformed using the log of the counts per million (CPM), and library size was normalized using calcNormFactors function with the method relative log expression (RLE).

### Gene set enrichment of DDEGs.

Gene set enrichment analysis (GSEA) in this study was performed using clusterProfiler (63) R package, and enrichment comparison from multiple groups as visualized using the compareCluster function.

### Permutation testing for assessing differences between groups over time.

After fitting gene expression longitudinal profiles from each severity group with a LOESS smoothing spline, we binned the time variable into 100 time intervals and calculated the observed area ratio between 2 curves within each time interval. Next, we shuffled the severity group label on the gene expression longitudinal profiles and repeated the previous step to calculate the shuffled area ratio for each time interval. We iterated the shuffling step 1000 times. In this way, for each time interval, we calculated the *P* value using the empirical distribution from the permutation test. This permutation test module in TrendCatcher allows users to assess between group differences of dynamic gene expression pathways in a time interval–dependent manner.

### Source code.

The R package source code of TrendCatcher is available on GitHub (https://github.com/jaleesr/TrendCatcher).

### Statistics.

The statistical tests for each computational approach model are described in-depth above. Generally, *P* values less than 0.05 were considered significant, and when multiple comparisons were performed, we applied Holm-Bonferroni method for *P* value adjustment.

### Study approval.

All the data sets used for the analyses were publicly available (as indicated in Table 1), and no human subjects were recruited.

## Author contributions

XW and JR designed the study, XW performed the analyses and wrote the first draft of the manuscript, JR revised the initial draft of the manuscript, and MAS and YD provided critical input for the analyses, visualizations, and revisions of the manuscript.

## Supplementary Material

Supplemental data

## Figures and Tables

**Table 1:**
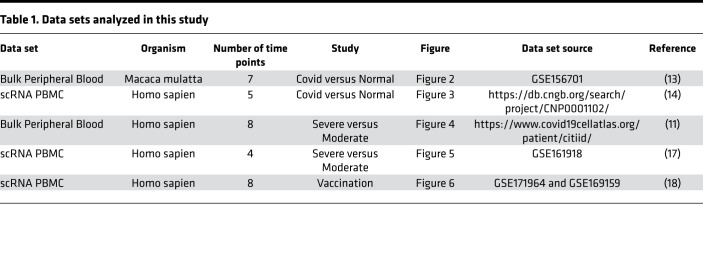
Data sets analyzed in this study

## Version 1 02/17/2022

In-Press Preview

## Version 2 04/08/2022

Electronic publication

## References

[1] Bar-Joseph Z (2012). Studying and modelling dynamic biological processes using time-series gene expression data. Nat Rev Genet.

[2] Hwang B (2018). Single-cell RNA sequencing technologies and bioinformatics pipelines. Exp Mol Med.

[3] Love MI (2014). Moderated estimation of fold change and dispersion for RNA-seq data with DESeq2. Genome Biol.

[4] McCarthy DJ (2012). Differential expression analysis of multifactor RNA-Seq experiments with respect to biological variation. Nucleic Acids Res.

[5] Ritchie ME (2015). limma powers differential expression analyses for RNA-sequencing and microarray studies. Nucleic Acids Res.

[6] Fischer DS (2018). Impulse model-based differential expression analysis of time course sequencing data. Nucleic Acids Res.

[7] Nueda MJ (2014). Next maSigPro: updating maSigPro bioconductor package for RNA-seq time series. Bioinformatics.

[8] Zhou F (2020). Clinical course and risk factors for mortality of adult inpatients with COVID-19 in Wuhan, China: a retrospective cohort study. Lancet.

[9] Wu ZY, McGoogan JM (2020). Characteristics of and important lessons from the Coronavirus Disease 2019 (COVID-19) outbreak in China Summary of a Report of 72 314 Cases From the Chinese Center for Disease Control and Prevention. JAMA.

[10] Pedersen SF, Ho YC (2020). SARS-CoV-2: a storm is raging. J Clin Invest.

[11] Bergamaschi L (2021). Longitudinal analysis reveals that delayed bystander CD8+ T cell activation and early immune pathology distinguish severe COVID-19 from mild disease. Immunity.

[12] Bernardes JP (2020). Longitudinal multi-omics analyses identify responses of megakaryocytes, erythroid cells, and plasmablasts as hallmarks of severe COVID-19. Immunity.

[13] Aid M (2020). Vascular disease and thrombosis in SARS-CoV-2-infected rhesus macaques. Cell.

[14] Zhu LN (2020). Single-cell sequencing of peripheral mononuclear cells reveals distinct immune response landscapes of COVID-19 and influenza patients. Immunity.

[15] Wang X (2019). Bulk tissue cell type deconvolution with multi-subject single-cell expression reference. Nat Commun.

[16] Combes AJ (2021). Global absence and targeting of protective immune states in severe COVID-19. Nature.

[17] Liu C (2021). Time-resolved systems immunology reveals a late juncture linked to fatal COVID-19. Cell.

[18] Arunachalam PS (2021). Systems vaccinology of the BNT162b2 mRNA vaccine in humans. Nature.

[19] Hao YH (2021). Integrated analysis of multimodal single-cell data. Cell.

[20] Aran D (2019). Reference-based analysis of lung single-cell sequencing reveals a transitional profibrotic macrophage. Nat Immunol.

[21] Krieger G (2020). Independent evolution of transcript abundance and gene regulatory dynamics. Genome Res.

[22] Yuan Y, Bar-Joseph Z (2019). Deep learning for inferring gene relationships from single-cell expression data. Proc Natl Acad Sci U S A.

[23] Graw F (2021). Deciphering the triad of infection, immunity and pathology. Elife.

[24] Van den Berge K (2020). Trajectory-based differential expression analysis for single-cell sequencing data. Nat Commun.

[25] Qiu XJ (2017). Reversed graph embedding resolves complex single-cell trajectories. Nat Methods.

[26] Chen G (2020). Clinical and immunological features of severe and moderate coronavirus disease 2019. J Clin Invest.

[27] Gong J (2020). Correlation analysis between disease severity and inflammation-related parameters in patients with COVID-19: a retrospective study. BMC Infect Dis.

[28] Mehta P (2020). COVID-19: consider cytokine storm syndromes and immunosuppression. Lancet.

[29] Qin C (2020). Dysregulation of immune response in patients with Coronavirus 2019 (COVID-19) in Wuhan, China. Clin Infect Dis.

[30] Goyal A (2020). Potency and timing of antiviral therapy as determinants of duration of SARS-CoV-2 shedding and intensity of inflammatory response. Sci Adv.

[31] Ackermann M (2021). Patients with COVID-19: in the dark-NETs of neutrophils. Cell Death Differ.

[32] Morrissey SM (2021). A specific low-density neutrophil population correlates with hypercoagulation and disease severity in hospitalized COVID-19 patients. JCI Insight.

[33] Cambier S (2022). Atypical response to bacterial co-infection and persistent neutrophilic broncho-alveolar inflammation distinguish critical COVID-19 from influenza. JCI Insight.

[34] Kaiser R (2021). Self-sustaining IL-8 loops drive a prothrombotic neutrophil phenotype in severe COVID-19. JCI Insight.

[35] Freire PP (2021). The relationship between cytokine and neutrophil gene network distinguishes SARS-CoV-2-infected patients by sex and age. JCI Insight.

[36] Libby P, Luscher T (2020). COVID-19 is, in the end, an endothelial disease. Eur Heart J.

[37] Bardoel BW (2014). The balancing act of neutrophils. Cell Host Microbe.

[38] Metzemaekers M (2021). Kinetics of peripheral blood neutrophils in severe coronavirus disease 2019. Clin Transl Immunology.

[39] Papayannopoulos V (2018). Neutrophil extracellular traps in immunity and disease. Nat Rev Immunol.

[40] Zuo Y (2021). Autoantibodies stabilize neutrophil extracellular traps in COVID-19. JCI Insight.

[41] Zuo Y (2020). Neutrophil extracellular traps in COVID-19. JCI Insight.

[42] Merad M, Martin JC (2020). Pathological inflammation in patients with COVID-19: a key role for monocytes and macrophages. Nat Rev Immunol.

[43] Grant RA (2021). Circuits between infected macrophages and T cells in SARS-CoV-2 pneumonia. Nature.

[44] Ackermann M (2020). Pulmonary vascular endothelialitis, thrombosis, and angiogenesis in Covid-19. N Engl J Med.

[45] Rapkiewicz AV (2020). Megakaryocytes and platelet-fibrin thrombi characterize multi-organ thrombosis at autopsy in COVID-19: A case series. EClinicalMedicine.

[46] Schmaier AA (2021). Tie2 activation protects against prothrombotic endothelial dysfunction in COVID-19. JCI Insight.

[47] Del Valle DM (2020). An inflammatory cytokine signature predicts COVID-19 severity and survival. Nat Med.

[48] Leisman DE (2020). Cytokine elevation in severe and critical COVID-19: a rapid systematic review, meta-analysis, and comparison with other inflammatory syndromes. Lancet Respir Med.

[49] Skendros P (2020). Complement and tissue factor-enriched neutrophil extracellular traps are key drivers in COVID-19 immunothrombosis. J Clin Invest.

[50] Carfi A (2020). Persistent symptoms in patients after acute COVID-19. JAMA.

[51] Huang CL (2021). 6-month consequences of COVID-19 in patients discharged from hospital: a cohort study. Lancet.

[52] Nalbandian A (2021). Post-acute COVID-19 syndrome. Nat Med.

[53] McNab F (2015). Type I interferons in infectious disease. Nat Rev Immunol.

[54] Muller U (1994). Functional role of type I and type II interferons in antiviral defense. Science.

[55] Blanco-Melo D (2020). Imbalanced host response to SARS-CoV-2 drives development of COVID-19. Cell.

[56] Hadjadj J (2020). Impaired type I interferon activity and inflammatory responses in severe COVID-19 patients. Science.

[57] Darazam IA (2021). Role of interferon therapy in severe COVID-19: the COVIFERON randomized controlled trial. Sci Rep.

[58] Loftus RM, Finlay DK (2016). Immunometabolism: cellular metabolism turns immune regulator. J Biol Chem.

[59] O’Neill LAJ (2016). A guide to immunometabolism for immunologists. Nat Rev Immunol.

[60] O’Brien KL, Finlay DK (2019). Immunometabolism and natural killer cell responses. Nat Rev Immunol.

[61] Gu C (2014). Smoothing spline ANOVA models: R package gss. J Stat Softw.

[62] Metwally AA (2018). MetaLonDA: a flexible R package for identifying time intervals of differentially abundant features in metagenomic longitudinal studies. Microbiome.

[63] Yu G (2012). clusterProfiler: an R package for comparing biological themes among gene clusters. OMICS.

[64] Kuleshov MV (2016). Enrichr: a comprehensive gene set enrichment analysis web server 2016 update. Nucleic Acids Res.

[65] Gu ZG (2016). Complex heatmaps reveal patterns and correlations in multidimensional genomic data. Bioinformatics.

