# Supplementary material for: Temporal transcriptomic analysis using TrendCatcher identifies early and persistent neutrophil activation in severe COVID-19
Source: JCI Insight. 2022 Apr 8;7(7):e157255. doi: 10.1172/jci.insight.157255 (PMC9057597; doi:10.1172/jci.insight.157255)
Supplement: Supplemental data [file jciinsight-7-157255-s231.pdf]

Supplemental material

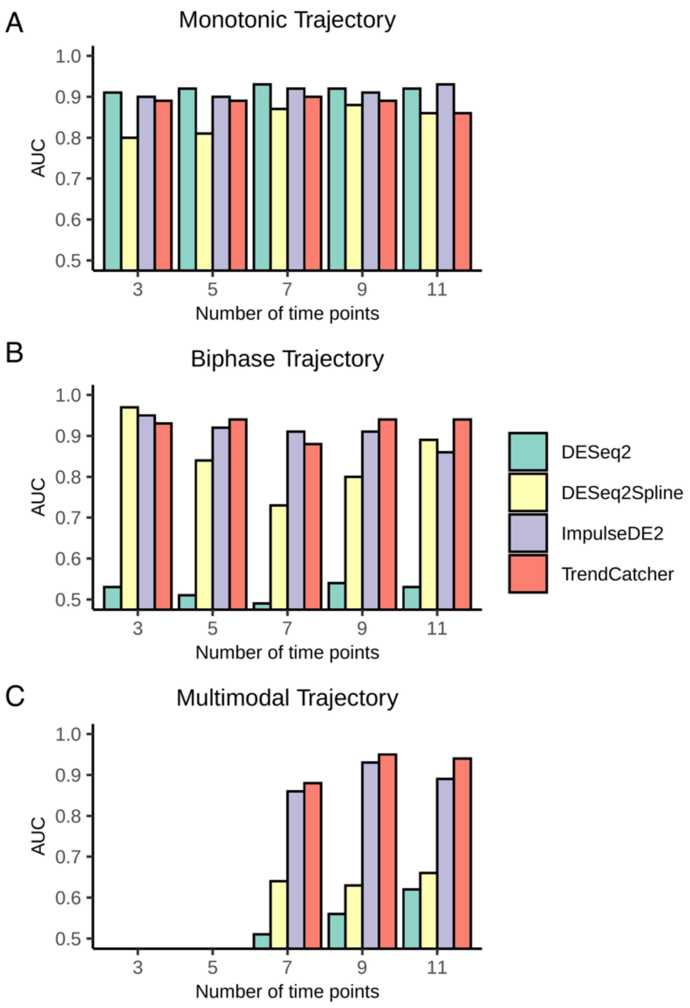

Supplemental Figure 1. TrendCatcher's prediction performance on different types of trajectories. (**A**) Prediction performance of TrendCatcher across varying numbers of time points for monotonic trajectories. (**B**) Prediction performance of TrendCatcher across varying numbers of time points for biphasic trajectories. (**C**) Prediction performance of TrendCatcher across varying numbers of time points for multimodal trajectories. DESeq2 is shown in green, DESeq2Spline is shown in yellow, ImpulseDE2 is shown in purple, TrendCatcher is shown in red. The x-axis represents number of time points from time course RNA-seq studies. The y-axis represents AUC values.

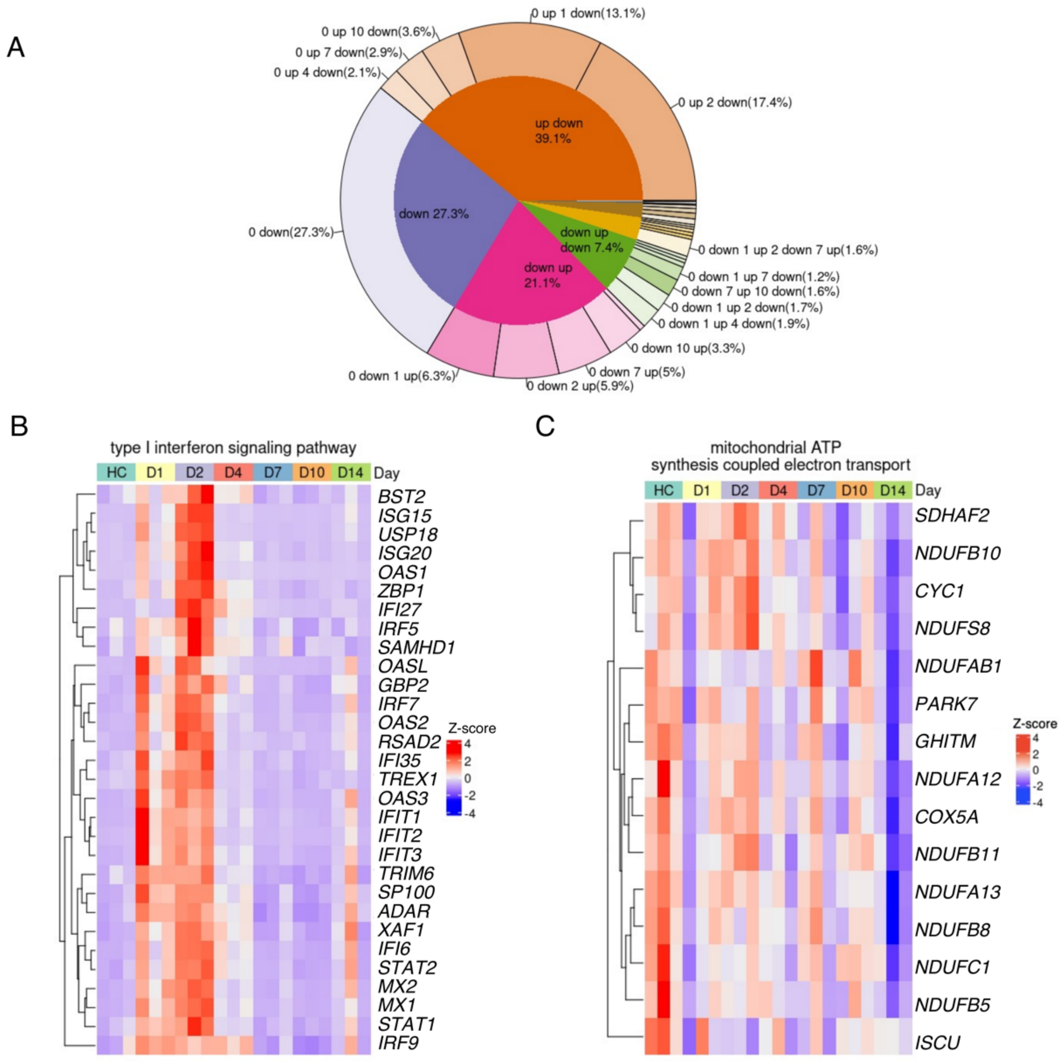

Supplemental Figure 2. Trajectory pattern composition of DDEGs and dynamic gene signatures from highlighted pathways. (**A**) Hierarchical pie chart shows the composition of trajectory patterns of DDEGs identified in a non-human primate bulk peripheral blood mRNA (Aid et al.). (**B**) Heatmap shows the dynamic gene signatures for SARS-CoV-2 infection from type I interferon signaling pathway. (**C**) Heatmap shows the dynamic gene signatures response for SARS-CoV-2 infection from mitochondrial ATP synthesis coupled electron transport pathway. Each row represents a gene signature, each column represents a sample. Samples were grouped by collecting time and marked by different colors at the top of heatmap. Color of the heatmap represents normalized z-score for gene expression.

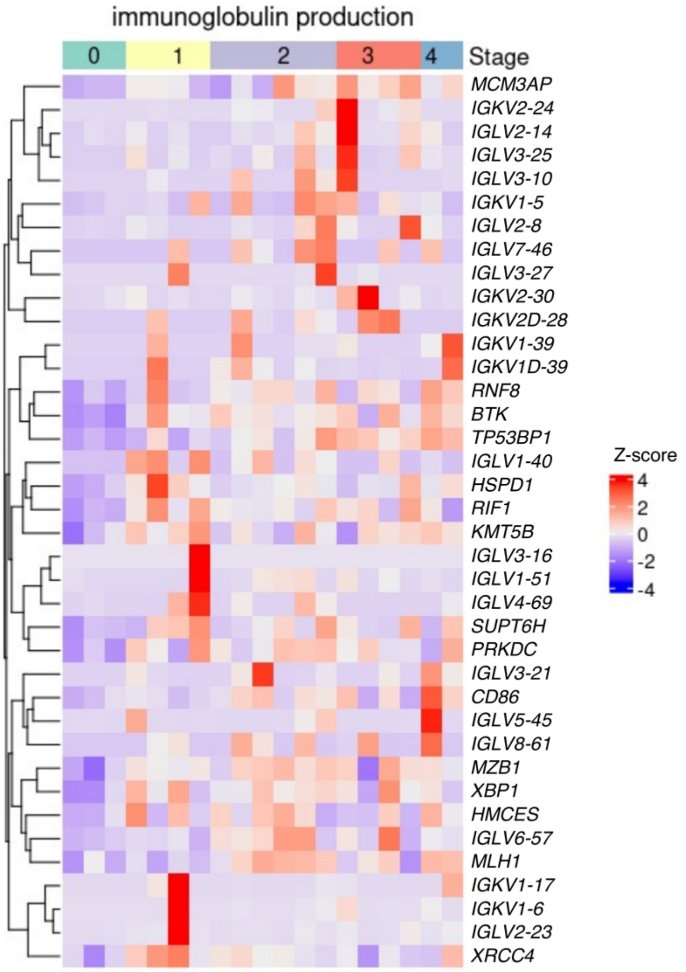

Supplemental Figure 3. Dynamic gene signatures of immunoglobulin pathway. Heatmap showing B cell's 38 DDEGs identified from immunoglobulin production process. Each row represents a gene signature, each column represents a sample. Samples were grouped by collecting time and marked by different colors at the top of heatmap. Color of the heatmap represents normalized z-score for gene expression.

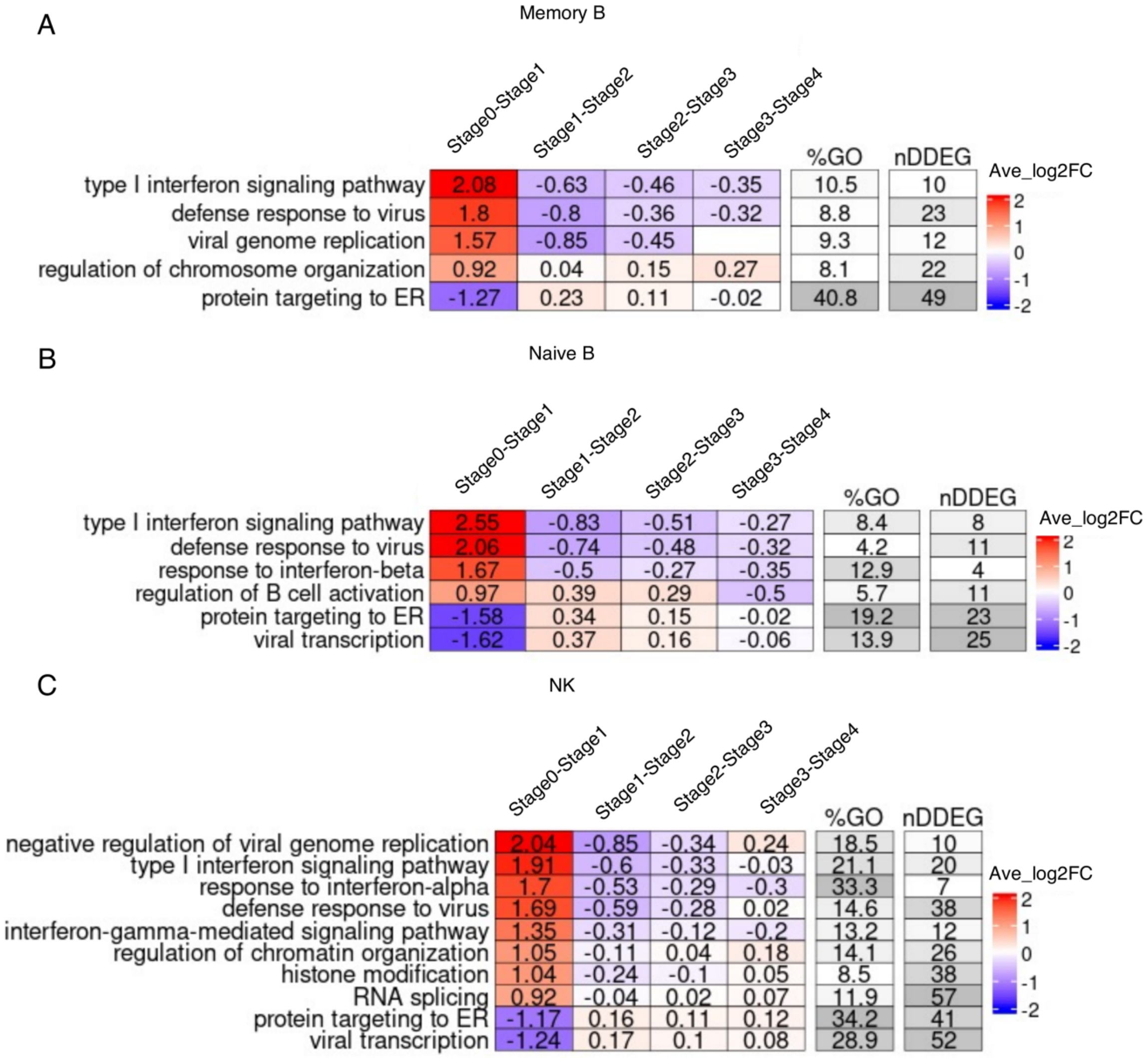

Supplemental Figure 4. TimeHeatmap of memory B cells, naive B cells and NK cells. (**A-C**) TimeHeatmap of (**A**) memory B, (**B**) naive B and (**C**) NK cells. Each column represents a time window. Stage 0 represents uninfected baseline. The “%GO” column represents the percentage of DDEGs found in the corresponding pathway. The “nDDEG” column represents number of DDEGs found in the corresponding pathway. Number in each grid presents the averaged log2 fold change of gene expressions compared to the previous time window. Color represents the averaged log2 fold-change of the DDEGs within each time window for the corresponding pathway.

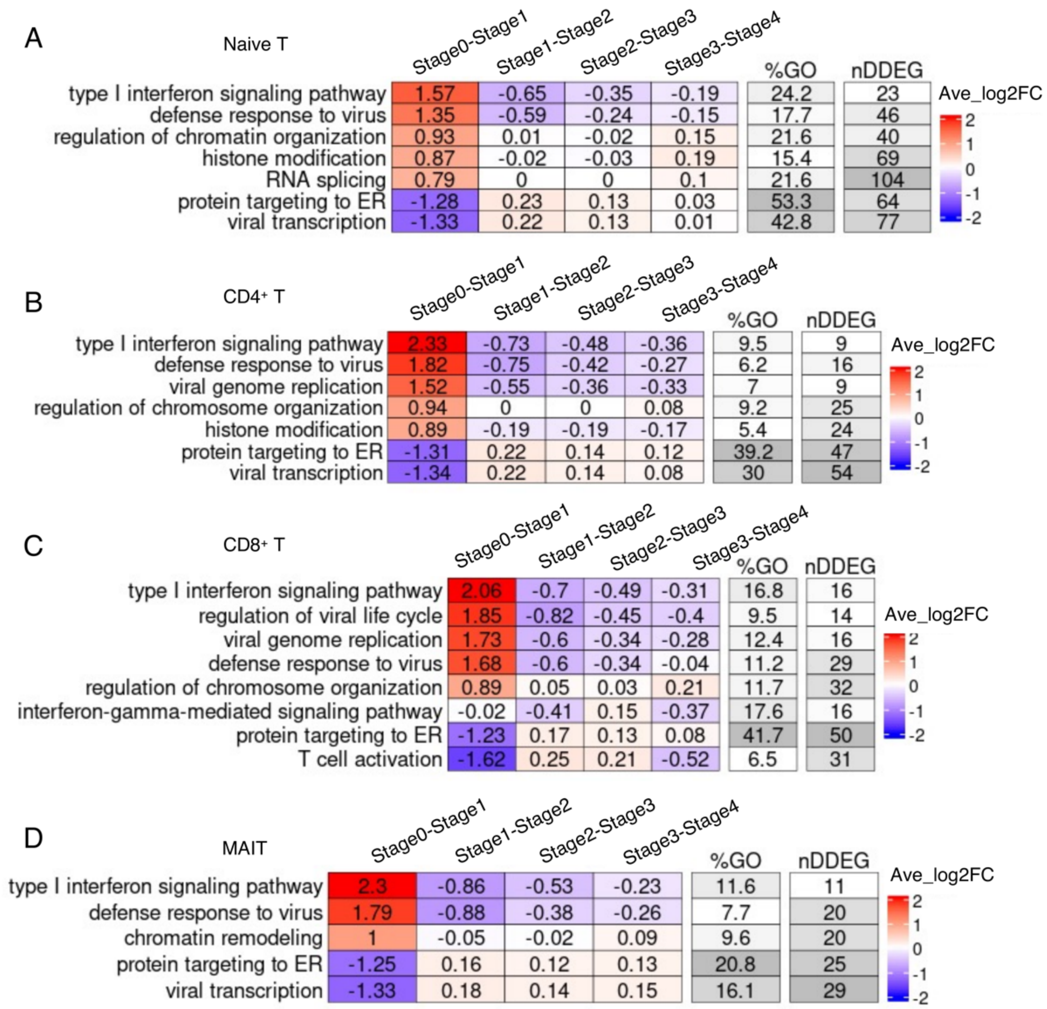

Supplemental Figure 5. TimeHeatmap of Naive T cell, $CD4^+$ T cell, $CD8^+$ T cell and MAIT cell. (**A-D**) TimeHeatmap of (**A**) Naive T cells, (**B**) $CD4^+$ T cells, (**C**) $CD8^+$ T cells and (**D**) MAIT cells. Each column represents a time window. Stage 0 represents uninfected baseline. The "%GO" column represents the percentage of DDEGs found in the corresponding pathway. The "nDDEG" column represents number of DDEGs found in the corresponding pathway. Number in each grid presents the averaged log2 fold change of gene expressions compared to the previous time window. Color represents the averaged log2 fold-change of the DDEGs within each time window for the corresponding pathway.

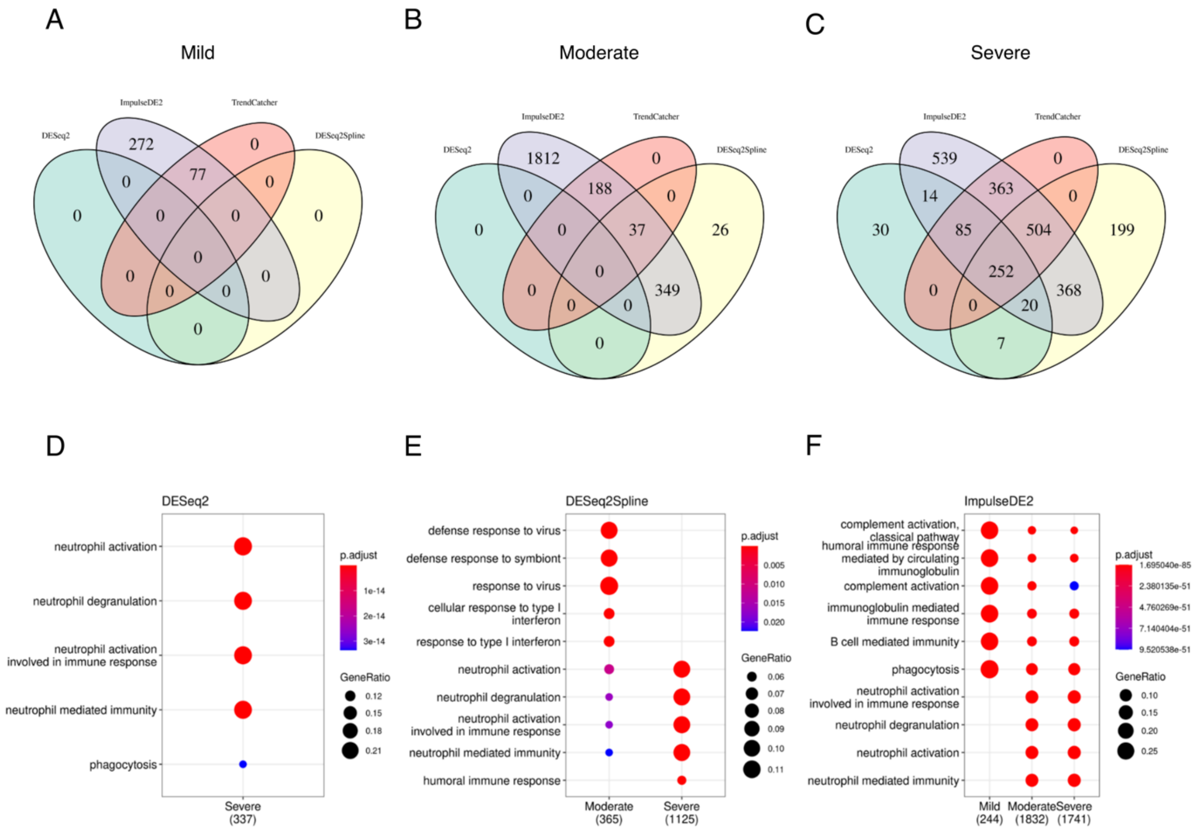

Supplemental Figure 6. Comparison of DDEGs identified by distinct computational platforms. (**A-C**) Venn diagrams show the number of DDEGs identified by DESeq2, DESeq2Spline, ImpulseDE2 and TrendCatcher in whole blood RNA-seq data from three different COVID-19 severity groups. (**A**) Mild COVID-19, (**B**) moderate COVID-19 and (**C**) severe COVID-19. (**D-F**) Comparison dot plots show the top GO enrichment result from DDEGs identified across three severity groups using DESeq2, DESeq2Spline and ImpulseDE2. (**D**) DESeq2, (**E**) DESeq2Spline and (**F**) ImpulseDE2. The x-axis represents comparison groups with the number of DDEGs shown in the brackets. The y-axis represents the enriched GO terms. P-adjust values are generated by Fisher's exact test and corrected using the Holm-Bonferroni method. Dot size represents the gene ratio.

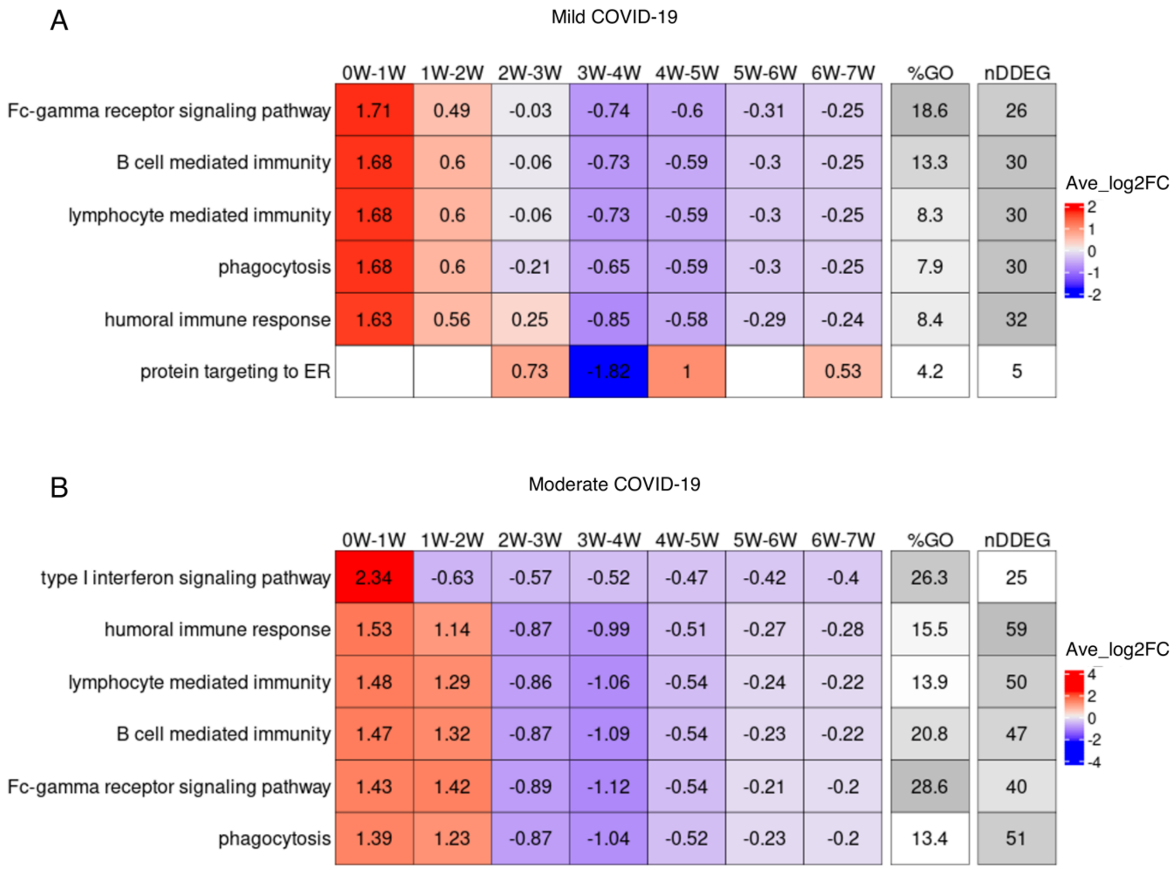

Supplemental Figure 7. TimeHeatmap of mild and moderate COVID-19. (**A**) TimeHeatmap of top dynamic GO terms found in severe COVID-19 shown in mild group. (**B**) TimeHeatmap of top dynamic GO terms found in severe COVID-19 shown in moderate group. Each column represents a time window. The "%GO" column represents the percentage of DDEGs found in the corresponding pathway. The "nDDEG" column represents number of DDEGs found in the corresponding pathway. Number in each grid presents the averaged log2 fold change of gene expressions compared to the previous time window. Color represents the averaged log2 fold-change of the DDEGs within each time window for the corresponding pathway.

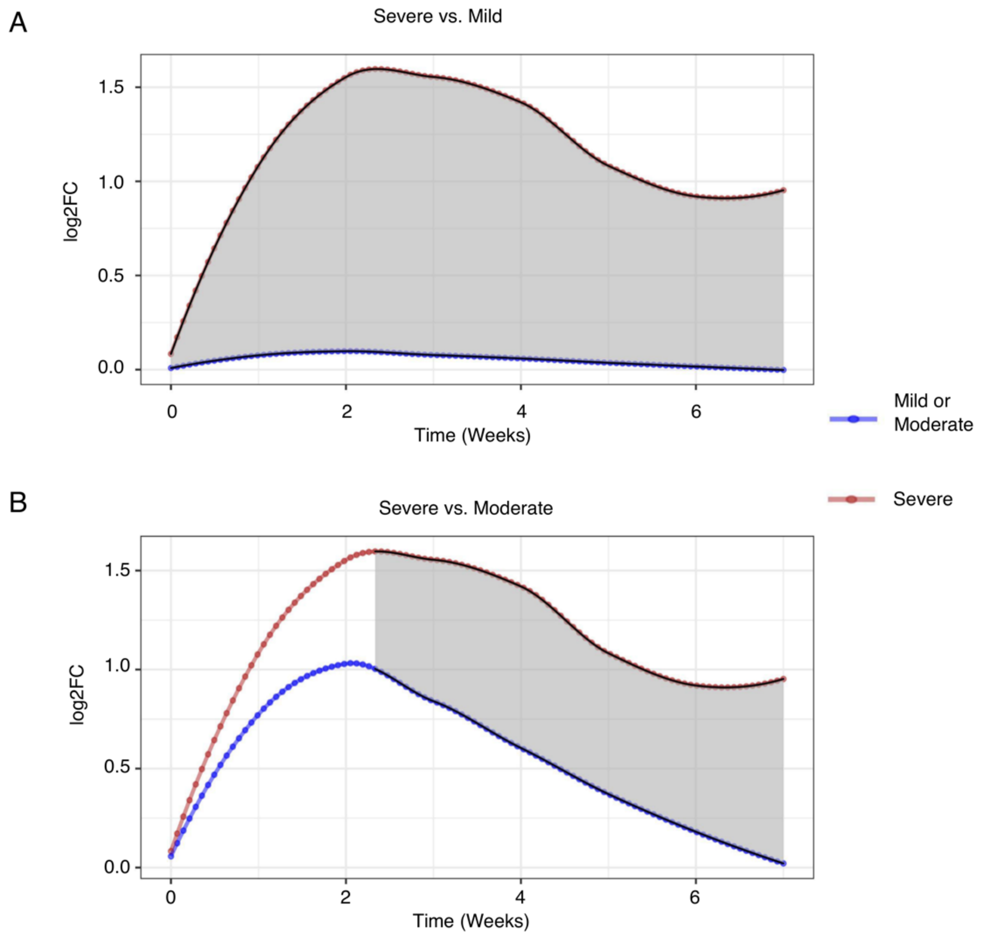

Supplemental Figure 8. Permutation testing for assessing differences over time between groups. (**A**) The permutation test shows significant separation between the mild and severe COVID-19 groups' LOESS fitting curves as early as the first week. (**B**) The permutation test shows that significant separation between the moderate and severe groups' LOESS fitting starts after week 2. Grey colored area means significant curve separation with P value less than 0.05. Blue curve represents mild group in (**A**) and moderate group in (**B**), red curve represents severe COVID-19 group. The x-axis is the time in the unit of week. The y-axis is the averaged log2 fold gene expression change over time compared to baseline.

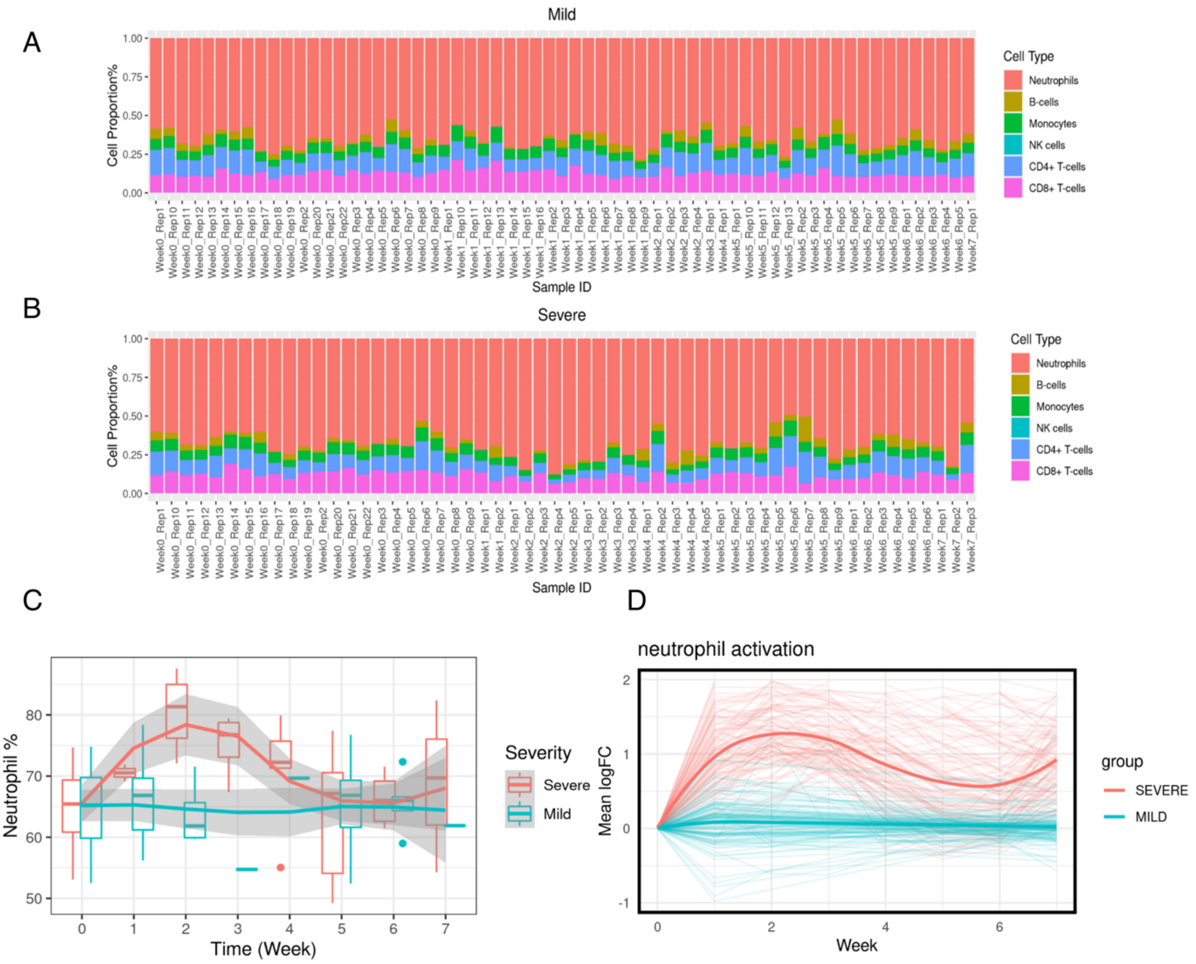

Supplemental Figure 9. Deconvolution of whole blood bulk RNA-seq dataset. (**A and B**) Stacked bar plot of estimated cellular percentages across patients at different time points in patients with mild and severe COVID-19. (**C**) Neutrophil percentage over time in patients with mild and severe COVID-19. X-axis depicts time in weeks. Y-axis depicts the neutrophil percentage using deconvolution of whole blood. (**D**) LOESS curve fitting using DDEGs of the neutrophil activation pathway enriched in deconvoluted neutrophils. Y-axis shows the averaged log2 fold change of gene expression over time compared to baseline. Red represents severe COVID-19 patients, blue represents mild COVID-19 patients.

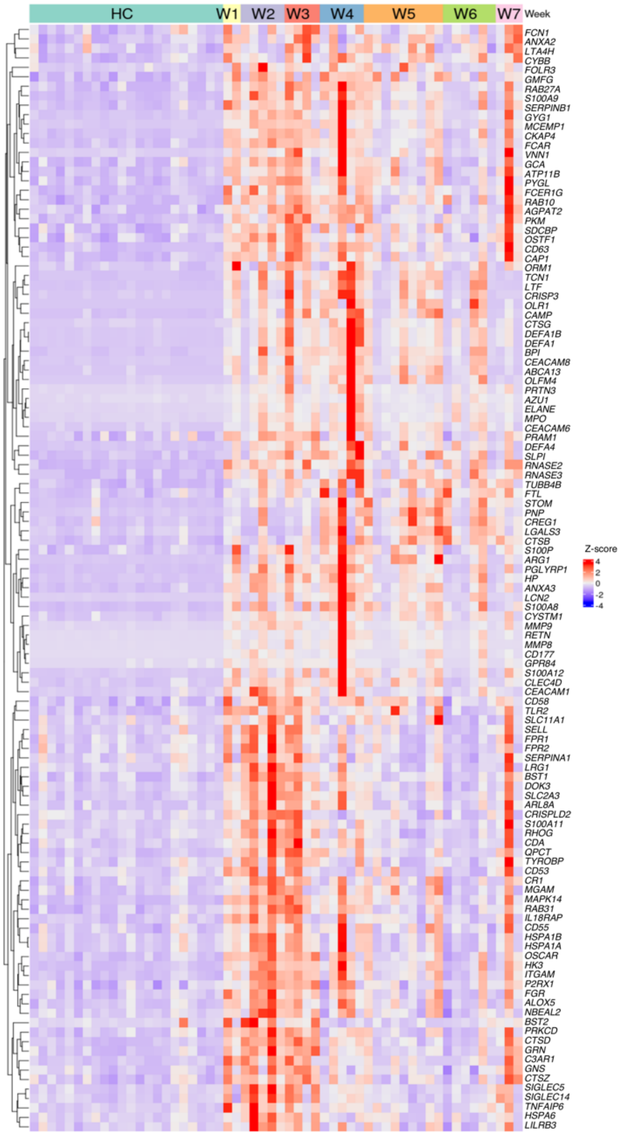

Supplemental Figure 10. Dynamic gene signatures of neutrophil activation from severe COVID-19. Heatmap of severe COVID-19 DDEGs identified from the neutrophil activation pathway. Each row represents a gene signature, each column represents a sample. Samples were grouped by collecting time and marked by different colors at the top of heatmap. Color of the heatmap represents normalized z-score for gene expression.

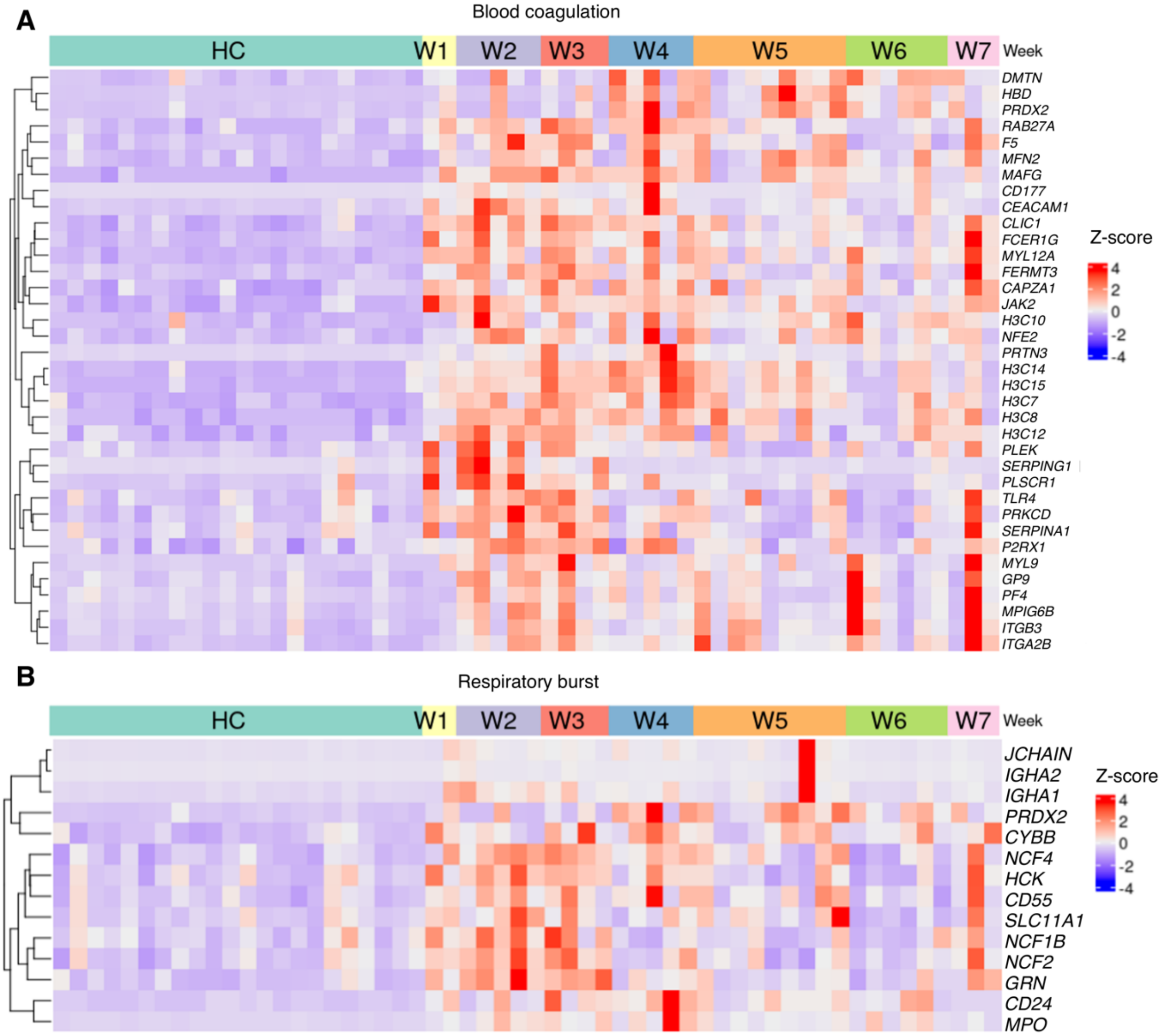

Supplemental Figure 11. Dynamic gene signatures of blood coagulation and respiratory burst pathways from severe COVID-19. (**A**) Heatmap of severe COVID-19 DDEGs identified from the blood coagulation pathway. (**B**) Heatmap of severe COVID-19 DDEGs identified from the respiratory burst pathway. Each row represents a gene signature, each column represents a sample. Samples were grouped by collecting time and marked by different colors at the top of heatmap. Color of the heatmap represents normalized z-score for gene expression.

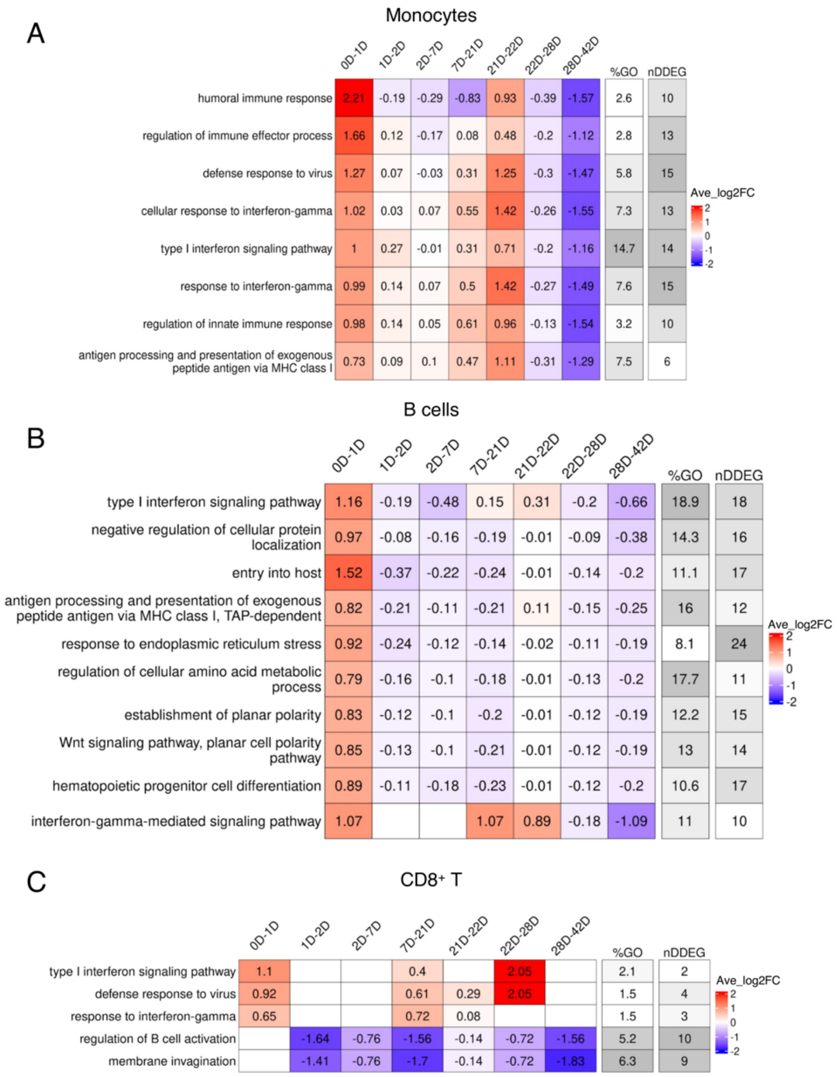

Supplemental Figure 12. TimeHeatmap of monocytes, B cells and CD8+ T cells. (**A**) TimeHeatmap of monocytes. (**B**) TimeHeatmap of B cells. (**C**) TimeHeatmap of $CD8^{+}$ T cells. day 1 is the first dose of vaccination, day 21 is the second dose of vaccination. Each column represents a time window. The “%GO” column represents the percentage of DDEGs found in the corresponding pathway. The “nDDEG” column represents number of DDEGs found in the corresponding pathway. Number in each grid presents the averaged log2 fold change of gene expressions compared to the previous time window. Color represents the averaged log2 fold change of the DDEGs within each time window for the corresponding pathway.

Supplemental Table 1. Number of DDEGs for each innate immune cell type identified by TrendCatcher.

NK Moderate	NK Severe	Monocyte Moderate	Monocyte Severe	DC Moderate	DC Severe
708	1,179	1,479	1,845	22	17

Supplemental Table 2. Number of DDEGs for each adaptive immune cell type identified by TrendCatcher.

B Moderate	B Severe	$CD4^+$ T Moderate	$CD4^+$ T Severe	$CD8^+$ T Moderate	$CD8^+$ T Severe	MAIT Moderate	MAIT Severe
969	1,344	190	1,060	634	817	71	13
